# Adhesive-Electrocoupling Hydrogels for Tissue Regeneration: Design, Mechanisms, and Perspectives

**DOI:** 10.34133/research.1296

**Published:** 2026-06-02

**Authors:** Jialiang Zhao, Ying Chen, Meilin Zuo, Xiong Lu, Chaoming Xie

**Affiliations:** Institute of Biomedical Engineering, College of Medicine, Southwest Jiaotong University, Chengdu, Sichuan 610031, China.

## Abstract

Endogenous bioelectric signals serve as pivotal physiological cues that govern cellular behavior and tissue regeneration. Conductive hydrogels have become a disruptive platform in the field of tissue engineering because they can promote tissue repair by utilizing endogenous electrical signals. Conventional conductive hydrogels often suffer from weak tissue adhesion and high contact impedance, which sever the continuity of endogenous bioelectric signals essential for regeneration. To bridge this gap, polyphenol-based adhesive-electrocoupling hydrogels have garnered increasing attention. The conductive network within adhesive-electrocoupling hydrogels facilitates electron transfer-mediated polyphenol redox cycling, preserving catechols for robust adhesion. This strong tissue interface integration maintains electrical signal transduction, constructing a conductive-adhesive synergistic circuit. This review first elucidates the biological effects of bioelectricity, establishing a theoretical foundation for conductive hydrogels to promote tissue repair using endogenous electrical signals. Subsequently, this review systematically summarizes the design strategies for adhesive-electrocoupling hydrogels mediated by polyphenol redox interactions, grounded in electron transfer mechanisms. Crucially, this review introduces the distinct biological mechanisms driving regeneration, highlighting the synergistic interplay among the intrinsic bioactivity of polyphenols, the modulation of cell behavior through endogenous electric field coupling, and cell adhesion. Furthermore, the versatile applications of adhesive-electrocoupling hydrogels in repairing electro-sensitive tissues are critically examined. Finally, this review discusses the current challenges and prospects.

## Introduction

The rapidly aging global population has precipitated a surge in chronic diseases, organ failure, and complex wounds, creating an urgent demand for advanced regenerative therapies. While tissue engineering offers a promising paradigm to circumvent the limitations of donor shortages and immune rejection, reconstructing the native tissue microenvironment remains a formidable challenge [1]. Bioelectricity constitutes a critical component of this physiological microenvironment [2]. Complex electric fields and currents exist in living organisms across multiple scales, ranging from transmembrane potentials and ion channel regulation to macroscopic electric fields at the tissue level, such as the “injury current” observed following skin damage [3]. These endogenous bioelectrical signals serve as pivotal cues governing fundamental cellular behaviors, including proliferation, apoptosis, migration, orientation, and differentiation [4]. Consequently, restoring the electrophysiological microenvironment is increasingly recognized as a decisive factor in regenerating electroactive tissues such as bone, periodontal, skin, myocardial, and brain tissues, as well as in accelerating wound healing [5].

Conductive hydrogels, which synergize the biomimetic features of hydrogels with the electrochemical characteristics of conductive materials, can highly mimic the electrophysiological microenvironment to facilitate tissue regeneration [6]. However, the instability of the conductive hydrogel–tissue interface represents a critical bottleneck limiting clinical translation. In the physiological environment, the ubiquitous hydration layer functions as a physical barrier, precluding intimate molecular contact between the hydrogel and the underlying tissue [7]. This interfacial separation precipitates 2 primary functional impairments: mechanical decoupling during dynamic tissue movements and signal attenuation caused by elevated interfacial impedance. Therefore, conductivity alone is insufficient. Stable, low-impedance interfacial wet adhesion is a prerequisite for effective bioelectrical signal transmission [8].

Integrating robust wet adhesion with high conductivity in a single hydrogel system presents a paradox, as most conductive fillers tend to compromise adhesion. However, nature provides an elegant solution to this dilemma through the adhesion mechanism of marine mussels [9]. Mussels maintain strong attachment to rocks in turbulent, wet environments primarily due to the secretion of adhesive proteins rich in 3,4-dihydroxyphenylalanine (DOPA), where the catechol group serves as the key functional motif [10]. Polyphenolic compounds, abundant in these functional groups, have emerged as ideal candidates to recapitulate this biological adhesion. Crucially, beyond their bioadhesive properties, polyphenols exhibit intrinsic redox activity [11]. They facilitate reversible interconversion between phenolic and quinone states via electron transfer in response to electrical stimuli, thereby achieving long-lasting adhesion [12,13]. This robust interfacial integration maintains electrical signal transduction, constructing a synergistic “adhesive-electrocoupling” circuit. Specifically, “adhesive-electrocoupling” represents a synergistic mechanism wherein adhesion and conductivity are mutually reinforcing. Within this framework, electron transfer through the conductive network drives the polyphenol redox cycle, regenerating catechol moieties to sustain durable adhesion. Reciprocally, the resulting stable adhesive interface minimizes interfacial impedance between the hydrogel and the tissue, ensuring the high-fidelity transmission of bioelectrical signals. Thus, polyphenol chemistry provides a versatile platform to integrate adhesion and electronics, enabling the construction of adhesive-electrocoupling hydrogels to achieve synergistic enhancement and promote tissue repair.

**Fig. 1.**
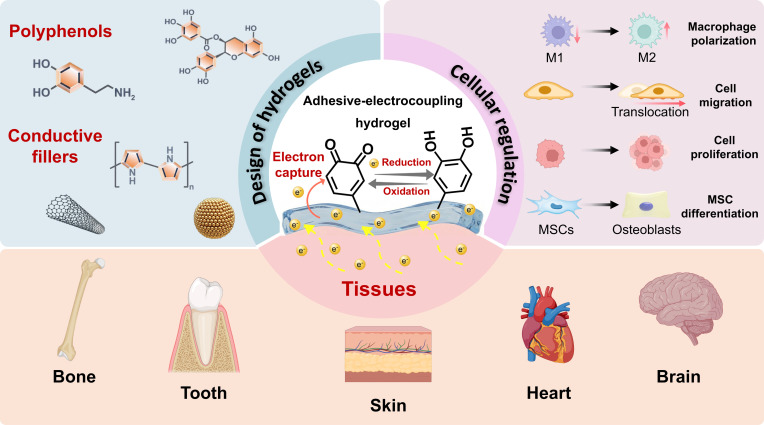
Adhesive-electrocoupling hydrogels and their tissue repair applications.

Previous reviews have predominantly focused on optimizing the conductivity or adhesion of hydrogels in isolation [14–17]. This review centers on polyphenol-mediated adhesive-electrocoupling effect. Specifically, this review investigates strategies to achieve a synergistic coupling of conductive and adhesive, detailing the applications of such hydrogels in tissue regeneration (Fig. 1).

This review first traces the historical development of bioelectricity and its biological effects, highlighting its pivotal role in tissue regeneration. Subsequently, this review outlines design strategies for polyphenol-mediated adhesive-electrocoupling hydrogels, illustrating how electron transfer mechanisms can be leveraged to preserve the catechol content and sustain long-term adhesion. It then elaborates on the biological mechanisms, establishing a foundation for clinical applications. Finally, the review summarizes the biomedical applications of these hydrogels and discuss future perspectives, identifying key opportunities and challenges for successful clinical translation.

## Bioelectricity

### History of bioelectricity

Bioelectricity refers to the electrical phenomena of life processes [18]. The study of bioelectricity dates back to 1789, when Luigi Galvani observed muscle twitching in frogs, proposing the concept of “animal electricity”. Although Alessandro Volta challenged this, attributing the phenomenon to “metallic electricity” from dissimilar metal contacts, Galvani subsequently validated the intrinsic biological nature of the current by inducing contractions via direct nerve–muscle contact without metals. Technological innovation in the 19th century propelled the field forward. Following Leopoldo Nobili’s development of the galvanometer, Carlo Matteucci detected currents in injured tissues and demonstrated that contracting muscles could stimulate adjacent nerves. Emil Heinrich du Bois-Reymond, the founder of modern electrophysiology, later identified the “negative variation”. Concurrently, Hermann von Helmholtz successfully measured nerve impulse propagation speed, refuting the prevailing belief that such signals were instantaneous.

Based on observation and data measurement, bioelectrical theory began to be gradually constructed and continuously improved. Using the differential rheotome, Julius Bernstein proposed the membrane theory, attributing resting potentials to selective K^+^ permeability. Upon stimulation, the membrane becomes more permeable to various cations, leading to depolarization. While Bernstein hypothesized that excitation collapsed the potential to zero, Charles Ernst Overton refined this model by identifying the critical role of Na^+^ exchange in generating potential overshoot. The modern era was defined by Alan Hodgkin and Andrew Huxley, who received the 1963 Nobel Prize for elucidating the ionic mechanisms of action potentials. Their work provided the theoretical bedrock for contemporary bioelectricity, which has evolved into a multidisciplinary field encompassing ion channel physiology, therapeutic electrical stimulation (ES), and bioelectronic medicine.

### Endogenous bioelectricity and electro-responsive repair

#### Bone

**Fig. 2.**
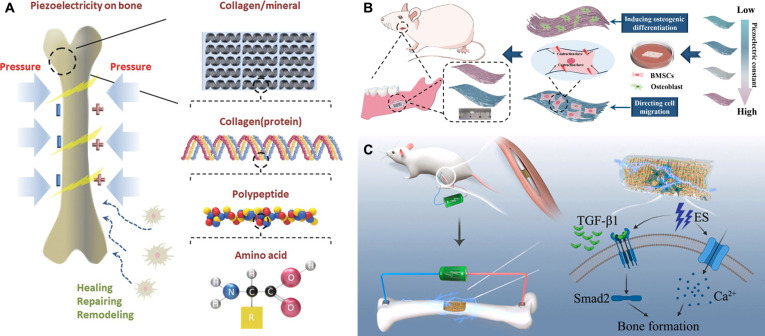
Bioelectricity in bone tissue. (A) Piezoelectric effect of bone. Reproduced with permission from [21]. Copyright 2020, Wiley-VCH. (B) Piezoelectric cellulose membrane for bone repair. Reproduced with permission from [32]. Copyright 2024, Oxford University Press. (C) Conductive hydrogels for bone repair. Reproduced with permission from [33]. Copyright 2023, Elsevier.

Bone tissue exhibits a high degree of structural organization and anisotropy. It is not a static structural support material but a dynamic, multifunctional living tissue that continuously senses and adapts to its mechanical environment. The ability of bone to perceive and respond to mechanical stimuli is fundamental to maintaining skeletal integrity and homeostasis. Within the complex cellular communication network of bone, endogenous bioelectrical signals generated through various electromechanical transduction mechanisms act as essential mediators of information exchange [19]. The dynamic adaptability of bone arises from its complex electrical properties, among which the piezoelectric effect is the most prominent [20]. Mechanical deformation of bone induces changes in endogenous electrical signals, which in turn regulate the activity of bone cells [21] (Fig. 2A). These bioelectrical signals play an indispensable role in bone formation, resorption, and repair, thereby influencing cellular behavior, tissue remodeling, and regenerative efficiency [22].

Piezoelectricity refers to the physical phenomenon in which mechanical stress induces charge separation within or on the surface of certain materials, generating an electric field. In 1957, Fukada and Yasuda first demonstrated the piezoelectric properties of bone tissue, establishing the foundation for research into the electrical functions of bone [23]. The piezoelectric effect of bone primarily arises from type I collagen molecules, which possess a highly ordered, noncentrosymmetric helical structure [24]. When subjected to mechanical forces such as tension or compression, collagen fibrils deform and induce charge redistribution, producing surface potential and ultimately giving rise to piezoelectric effects. The magnitude of bone’s piezoelectric response is regulated by several factors. First, the orientation of collagen fibrils plays a crucial role, and highly aligned fibrils in bone markedly enhance signal generation [25,26]. Second, the degree of mineralization affects the piezoelectric effect, as mineral crystals interact with the charge distribution of collagen molecules [27,28]. Third, the hydration state of the tissue influences the electrical properties of the collagen–mineral complex [29]. Studies have shown that piezoelectric coefficients vary among different bone sites; for example, the femur exhibits a coefficient of approximately 0.7 pC/N, whereas the tibia demonstrates substantially higher values ranging from 7.7 to 8.7 pC/N [30,31]. This property enables bone to continuously generate piezoelectric electrical signals during physiological movement through mechanical deformation. These signals, in turn, modulate the biological behaviors of bone-related cells through various regulatory mechanisms, including proliferation, differentiation, migration, matrix synthesis, and signal transduction [22].

In recent years, research on piezoelectric effect-based hydrogels for bone tissue repair has made remarkable progress. For instance, Chen et al. [32] fabricated poly(l-lactic acid) nanofiber membranes with gradient-tunable piezoelectric constants via directional electrospinning. These membranes enhanced bone marrow stromal cell (BMSC) proliferation, osteogenic differentiation, cell adhesion, migration, and morphological extension through piezoelectric stimulation mediated by endogenous mechanical forces, achieving complete restoration of the rat mandibular structure (Fig. 2B). Yu et al. [33] synthesized a novel nanoconductive hydrogel and combined it with exogenous ES to elevate endogenous transforming growth factor-β1 (TGF-β1) levels and activate the TGF-β/Smad2 signaling pathway, thereby promoting calcium deposition and bone formation (Fig. 2C). Li et al. [34] developed a 3-dimensional (3D)-printed piezoelectric hydrogel scaffold and used low-intensity pulsed ultrasound to activate its piezoelectric response, which enhanced intracellular Ca^2+^ influx and activated the phosphatidylinositol 3-kinase (PI3K)/Akt pathway, ultimately promoting osteoblast differentiation and bone matrix synthesis.

Collectively, these studies underscore the pivotal role of endogenous bioelectricity in maintaining bone homeostasis and validating the strategy of recapitulating the native electrical microenvironment for tissue repair. By leveraging the electromechanical coupling effects of piezoelectric materials, it becomes possible to translate mechanical stimuli into precise bioelectrical cues that modulate intracellular signaling pathways. Consequently, the integration of electroactivity into scaffold design represents a paradigm shift from purely structural support to functional bio-regulation, offering a robust platform for enhanced bone regeneration.

#### Skin

As the largest organ of the human body, the skin plays a central role in maintaining homeostasis and defense. It regulates body temperature, preserves water and electrolyte balance, mediates innate and adaptive immune responses, and senses external stimuli such as mechanical, thermal, and chemical stimulation [35]. Furthermore, as the primary barrier against physical injury, chemical insult, and microbial invasion, the skin is also one of the most vulnerable tissues in the body [36]. Skin injury can result from burns, mechanical trauma, chemical exposure, or pathological conditions.

**Fig. 3.**
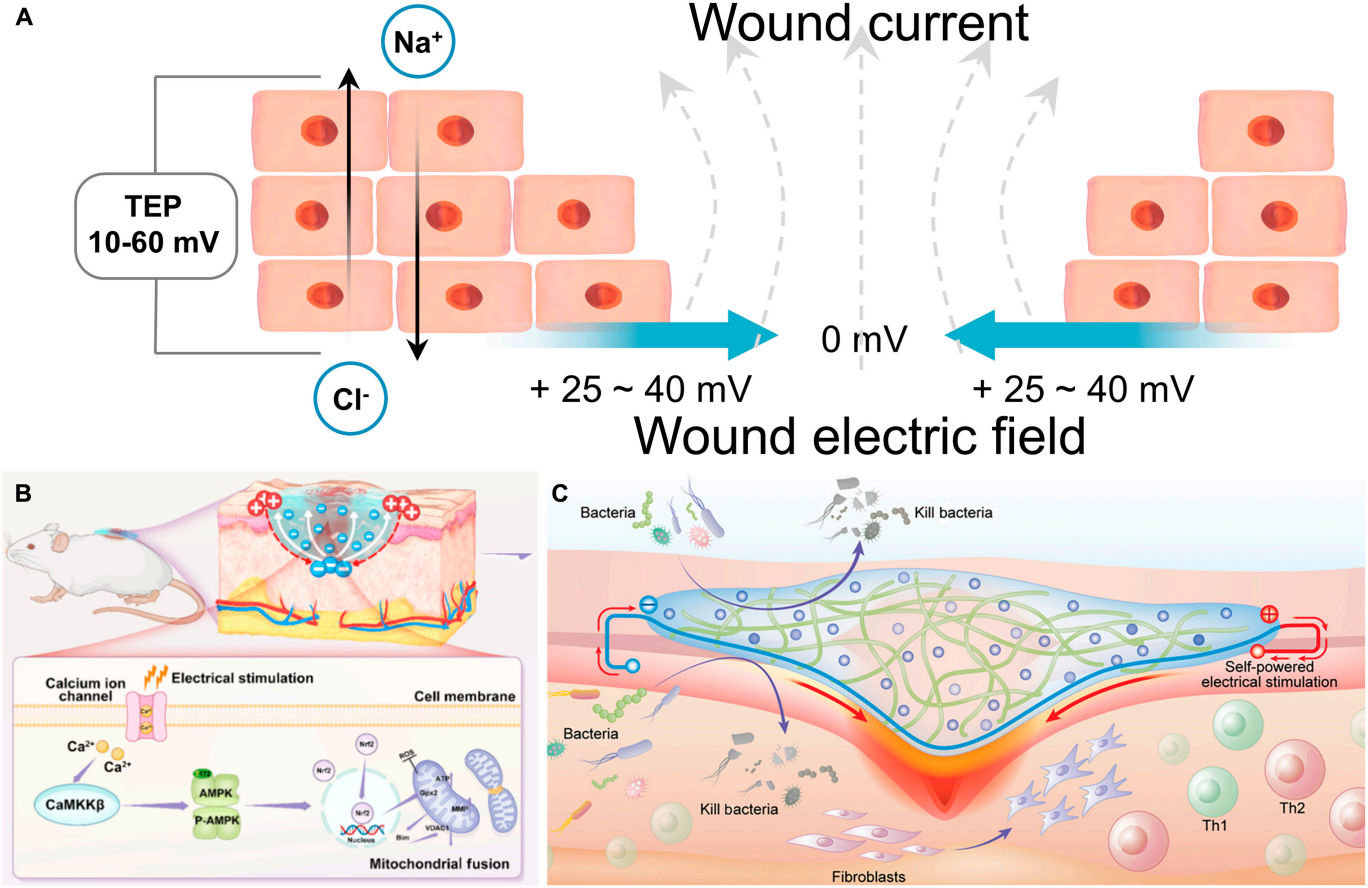
Bioelectricity in skin tissue. (A) TEP in skin tissue and endogenous electric field in skin wounds. (B) Self-powered hydrogel accelerates wound healing. Reproduced with permission from [45]. Copyright 2025, American Chemical Society. (C) Electroactive hydrogel accelerates wound healing. Reproduced with permission from [48]. Copyright 2025, Wiley-VCH.

Notably, a stable endogenous electric field exists within skin tissue, a property essential for both physiological function and wound repair [37]. In structurally intact skin, epidermal cells establish a transepithelial potential (TEP) through active ion transport, with an amplitude typically ranging from 10 to 60 mV [38] (Fig. 3A). When the skin is injured, the epidermal architecture is disrupted, disturbing the original ion gradient and markedly decreasing the TEP [39]. This disruption forms an endogenous wound electric field (WEF) between the intact epidermis and the wound site [3]. As early as the 19th century, the German physiologist Emil Du Bois-Reymond first documented natural electrical currents generated in human wounds, laying the foundation for subsequent research. Building upon this discovery, researchers proposed the “skin battery” theory, which posits that the strength of the WEF is closely correlated with both the distance from the wound edge and the wound surface area [38]. Specifically, when the epithelial barrier is compromised, the “battery system” formed by epidermal cells short-circuits, causing a sharp decrease in TEP. Consequently, the wound site becomes negatively charged relative to the surrounding intact skin, functioning as the cathode, whereas the undamaged area serves as the anode [40]. A stable potential gradient thus develops between the 2 regions, generating a lateral electric field of approximately 25 to 40 mV that drives current toward the wound site [3]. Further studies have revealed that Cl^−^, Na^+^, and K^+^ are the principal ionic carriers of this wound current, indicating that it essentially represents a directional ionic flow [41,42]. Therefore, ion pumps responsible for transmembrane transport play indispensable roles in maintaining the wound’s electrical potential and regulating WEF intensity.

Currently, accumulating evidence demonstrates that these endogenous bioelectrical signals are not only fundamental to maintaining skin homeostasis but also serve as core biophysical regulators of cellular behavior. Endogenous electric fields can direct cell migration, modulate proliferation, regulate apoptosis, and guide differentiation [43,44]. These findings provide critical theoretical and experimental support for understanding the electrophysiological mechanisms of wound healing and for developing novel treatment strategies involving conductive dressings and exogenous ES.

In recent years, numerous innovative studies have emerged in the field of wound healing technologies based on bioelectrical regulation. Qin et al. [45] developed a temperature-responsive electrostimulatory hydrogel wound dressing that amplifies the endogenous electric field through external ES, thereby promoting cell proliferation and migration. ES activates cellular Ca^2+^ channels, increasing intracellular Ca^2+^ levels and enhancing mitochondrial dynamics and angiogenesis via the Ca^2+^/CaMKKβ/AMPK/Nrf2 signaling pathway, ultimately accelerating wound healing (Fig. 3B). Wang et al. [46] designed an ultrasound-responsive, electrically stimulable conductive composite hydrogel based on interfacial ion–electron transfer. Upon ultrasound activation, ion transport at the fluid–solid interface generates localized ES, which promotes nerve growth factor secretion, angiogenesis, and fibroblast migration in diabetic wounds. Yang et al. [47] fabricated a biocompatible liquid gold composite wound dressing that combines electrical conductivity, high stretchability, and recyclability. The combined application of this dressing with ES facilitated fibroblast migration and stimulated the regeneration and healthy growth of hair follicles at the injury site, thereby reducing scarring and promoting high-quality wound healing. Liu et al. [48] constructed an electroactive hydrogel incorporating a conductive network capable of storing and releasing charge, enabling continuous transmission of bioelectrical signals to promote wound healing by modulating immune responses and reducing fibrosis (Fig. 3C).

Collectively, these advances highlight the critical function of the “skin battery” in orchestrating wound repair and validate the therapeutic potential of bioelectrical modulation. By bridging the disruption in the TEP, electroactive dressings do not merely serve as physical barriers but act as active physiological regulators that re-establish the endogenous electrical microenvironment. Whether through intrinsic conductivity or responsiveness to external stimuli, these materials effectively mobilize intracellular signaling to accelerate re-epithelialization and angiogenesis. Importantly, the ability of these strategies to modulate immune responses and promote skin appendage regeneration suggests a promising pathway toward achieving high-quality, scarless tissue restoration.

#### Heart

**Fig. 4.**
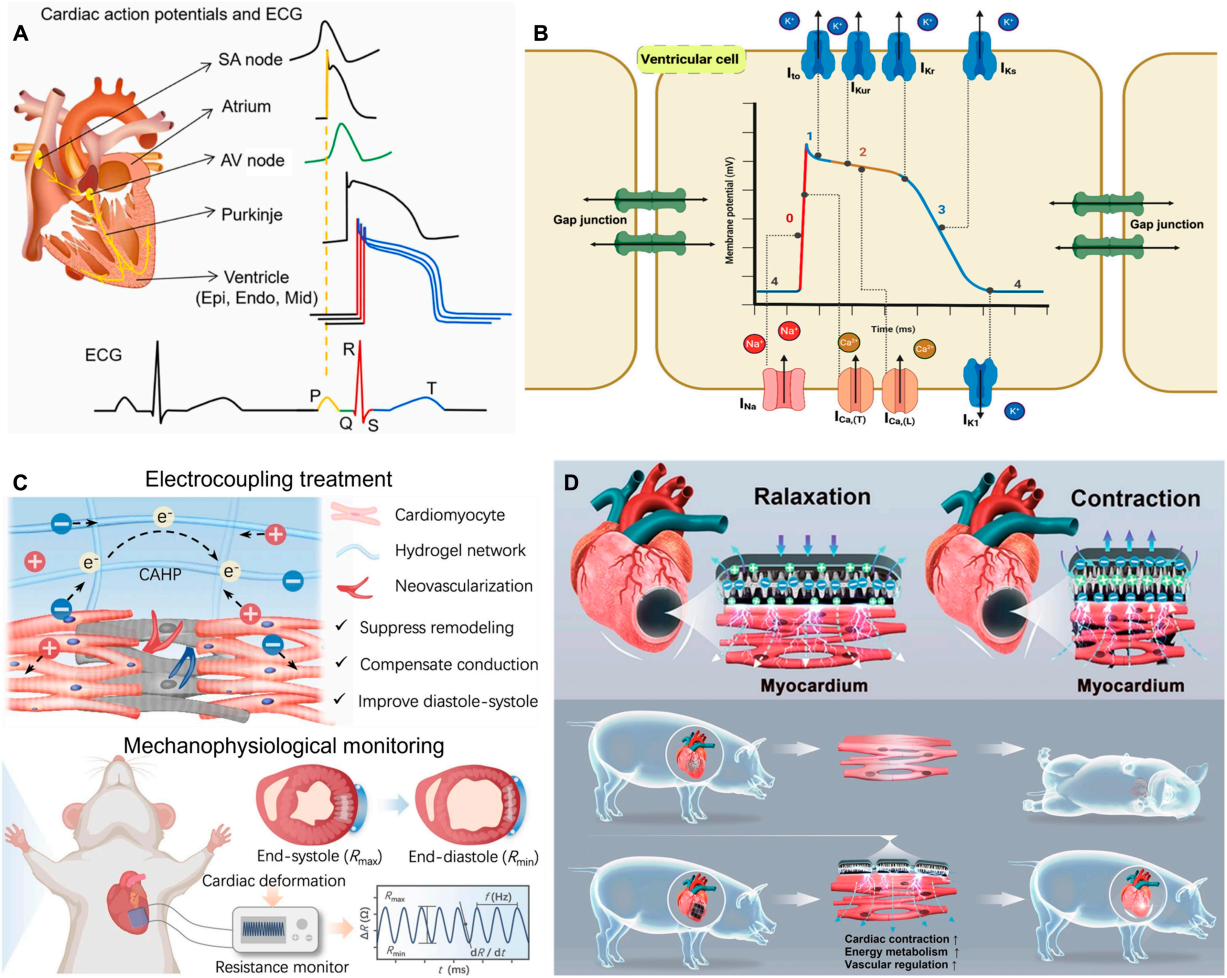
Bioelectricity in heart tissue. (A) Schematic cartoon to show cardiac action potentials from different regions of the heart, as well as the ECG. Reproduced with permission from [53]. Copyright 2023, Elsevier. (B) Heart action-potential phases and ion channel localization. Reproduced with permission from [54]. Copyright 2025, MDPI. (C) Integrated electrical coupling cardiac patch for treatment and monitoring. Reproduced with permission from [63]. Copyright 2023, Springer Nature. (D) Self-powered conductive patch for MI treatment. Reproduced with permission from [64]. Copyright 2024, Springer Nature.

Cardiovascular diseases (CVDs) are among the leading causes of death and disability worldwide [49]. Their high morbidity and mortality rates continue to rise, posing a serious threat to public health. The heart, the central powerhouse of the circulatory system, is composed of proteins, connective tissue, and cardiomyocytes (CMs). Its continuous and rhythmic mechanical contractions are essential for sustaining life. The normal pumping function of the heart relies on a highly coordinated electrical conduction system that synchronizes CM contraction to ensure efficient mechanical output [50]. Cardiac electrical signal generation and conduction follow a highly ordered pathway: Electrical impulses originate from pacemaker cells in the sinoatrial node, propagate through the atrioventricular node and Purkinje fiber network, and ultimately trigger ventricular CM contraction [51,52]. The overall action potential of the whole heart manifests as a cumulative electrical signal that projects onto body surface electrodes. Measured by the electrocardiogram (ECG), this surface projection serves as a proxy for monitoring cardiac electrophysiology. Specifically, the ECG records a distinct sequence of P, Q, R, S, T, and U waves, where each individual waveform reflects a particular phase of the cardiac cycle [53] (Fig. 4A). The coordinated contraction of the atria and ventricles depends on the rapid activity of multiple ion channels [54] (Fig. 4B). During this process, gap junctions between adjacent CMs form the structural basis for rapid action potential propagation, while desmosomes provide mechanical coupling, ensuring that myocardial tissue contracts and relaxes in a regular and synchronized manner, thereby maintaining effective blood circulation.

Among the broad spectrum of CVDs, myocardial infarction (MI) resulting from coronary artery occlusion represents a predominant source of morbidity and mortality, frequently cited as the leading cause of death within this pathological category [55]. Coronary ischemia and hypoxia directly induce CM apoptosis, tissue fibrosis, scar formation, and ventricular remodeling [56]. These pathological changes severely disrupt normal cardiac electrical signaling. In particular, the formation of nonconductive scar tissue interrupts the anisotropic propagation of electrical impulses, leading to impaired myocardial contraction and overall cardiac dysfunction. Therefore, restoring electrical signal transmission in damaged myocardial tissue remains a major challenge in cardiac repair and a central focus of emerging therapeutic strategies.

In recent years, substantial advances have been made in myocardial repair using conductive biomaterials [57,58]. Studies have shown that coculturing conductive patches with cells can enhance intercellular communication, improve cell viability, promote proliferation, and regulate cellular behavior [59]. Connexins play a pivotal role in forming gap junctions between CMs, with connexin 43 (CX-43) being particularly important. CX-43 maintains the stability of the cardiac conduction system by promoting synchronized action potential propagation, and its abnormal expression can directly induce arrhythmias or even fatal cardiac events [60]. Experimental studies have further shown that coculturing conductive materials with C2C12 myocytes promotes directional migration, myotube formation, and cell fusion and maturation [61]. Similarly, H9c2 CMs cocultured with conductive materials exhibited high viability and proliferation rates, providing experimental evidence supporting the application of conductive materials in myocardial repair [62]. Yu et al. [63] developed a functionalized polyaniline-based time-adhesive hydrogel patch with excellent electrochemical properties. It can sensitively sense microdeformations mediated by cardiac mechanophysiology, monitor the diastolic–systolic amplitude and rhythm of infarcted myocardium online, and effectively inhibit ventricular remodeling, promote angiogenesis, and improve electrophysiological function through electrocoupling therapy (Fig. 4C). Qiu et al. [64] designed a self-powered conductive cardiac patch capable of delivering ES to infarcted tissue. This system markedly accelerated excitation conduction between healthy and infarcted myocardium, restored the electrophysiological function of ischemic myocardial tissue, and achieved substantial myocardial repair in a miniature pig model of MI (Fig. 4D).

In conclusion, the development of conductive biomaterials addresses the critical challenge of electrical decoupling caused by post-infarction fibrosis. By effectively bridging the insulating scar tissue and up-regulating the expression of CX-43, these electroactive scaffolds re-establish a functional syncytium between healthy and damaged myocardium. This restoration of synchronous electrical propagation is paramount, as it not only improves mechanical cardiac output but also substantially mitigates the risk of lethal arrhythmias, paving the way for safer and more efficient myocardial regeneration strategies.

#### Nerve

Nerve tissue, comprising the central and peripheral nervous systems, plays an indispensable role in sensory processing, motor control, and higher cognitive functions [65]. However, neural tissue is highly vulnerable to trauma, neurodegenerative diseases, and metabolic disorders. Such damage not only leads to neuronal death and axonal transection but also directly disrupts the endogenous electrochemical signaling circuits essential for neural communication [66].

**Fig. 5.**
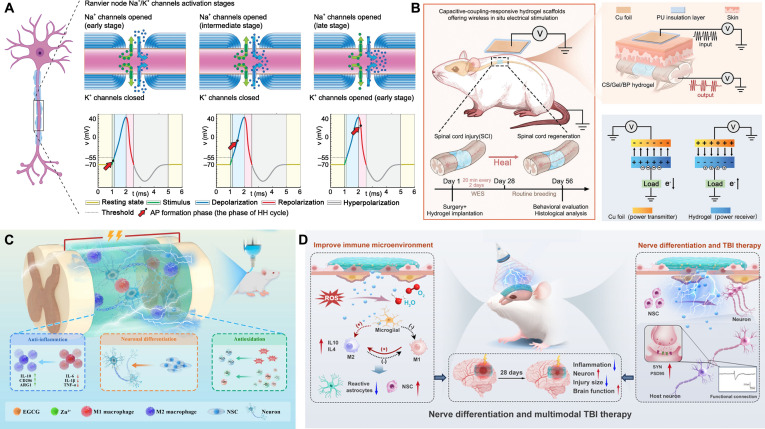
Bioelectricity in nerve tissue. (A) Schematic diagram of neuronal action potential generation. Reproduced with permission from [73]. Copyright 2022, Elsevier. (B) Capacitive-coupling-responsive hydrogel scaffolds for treating spinal cord injury. Reproduced with permission from [74]. Copyright 2024, Wiley-VCH. (C) Conductive hydrogels in conjunction with ES for treating spinal cord injury. Reproduced with permission from [75]. Copyright 2026, Springer Nature. (D) Piezoelectric hydrogels for treating traumatic brain injury. Reproduced with permission from [76]. Copyright 2025, Wiley-VCH.

Electrical signal transduction is a fundamental characteristic of the nervous system, central to information transmission, the regulation of synaptic plasticity, and the control of neurophysiological processes [67]. In recent years, various ES modalities, including deep brain, transcranial, and transcutaneous approaches, have emerged as effective strategies for neuromodulation and the promotion of neurogenesis [68,69]. Extensive preclinical and clinical studies have confirmed their efficacy in advancing neural repair, demonstrating outcomes such as induced neurogenesis in the adult mammalian hippocampus and substantially improved motor function in patients following neurological injury [70,71]. At the cellular level, the propagation of these electrical signals is highly dependent on the action potential [72]. In the resting state, the transmembrane resting potential of a typical neuron is maintained at approximately −70 mV. When a stimulus elevates the membrane potential to the threshold (approximately −55 mV), voltage-gated sodium channels rapidly open. This causes a massive influx of Na^+^, driving the membrane potential to instantaneously depolarize to around +30 mV. Subsequently, the delayed opening of K^+^ channels initiates membrane repolarization. During this phase, the potential typically drops briefly below the resting potential to approximately −90 mV, entering a state of hyperpolarization and a corresponding refractory period. This refractory phase not only temporarily prevents the neuron from responding to subsequent stimuli but also precludes the backward propagation of action potentials along the axon, thereby ensuring the efficient, unidirectional transmission of nerve signals. Finally, ion pumps act to restore the cell membrane to its initial resting potential [73] (Fig. 5A).

Traditional neural tissue engineering scaffolds, such as most natural or synthetic polymers, are typically electrically insulating. When implanted into sites of neuropathy or tissue defects, they provide physical support but act as insulators, obstructing the electrical signaling cascade between neurons. Driven by a deeper understanding of these electrophysiological mechanisms and the need to overcome the limitations of traditional biomaterials, the application of conductive biomaterials for neural tissue repair has witnessed groundbreaking progress in recent years [72]. Wu et al. [74] developed a biodegradable conductive hydrogel as an energy transfer medium for capacitively coupled wireless power generation. Under a high-frequency electric field, the in situ ES promoted neural stem cell (NSC) proliferation and neurotrophic factor secretion. In a complete spinal cord injury rat model, this scaffold substantially enhanced remyelination and axonal regeneration, accelerating functional recovery (Fig. 5B). Similarly, Du et al. [75] engineered a conductive hydrogel with tissue-matched conductivity to bridge the damaged electrophysiological network. This scaffold mitigates the inflammatory cascade by inducing anti-inflammatory M2 macrophage polarization and reducing neuronal apoptosis. When combined with exogenous ES, the hydrogel generates localized bioelectric cues that precisely drive endogenous NSC neuronal differentiation, accelerating neural circuit reconstruction (Fig. 5C). Liang et al. [76] designed an immunopiezoelectric hydrogel transducer that converts programmable ultrasound into localized ES. These in situ signals promote NSC differentiation into glutamatergic and γ-aminobutyric acid-ergic neurons, accelerating synaptic integration via the PI3K/Akt pathway. Furthermore, a polydopamine (PDA) coating enhances piezoelectric output, scavenges reactive oxygen species (ROS), and induces M2 microglial polarization. This bidirectional “immuno-electrical” synergy effectively reshapes the injury microenvironment, offering a robust paradigm for neural repair through wireless bioelectricity (Fig. 5D).

In conclusion, the inherent reliance of the nervous system on precise electrochemical signaling makes it profoundly vulnerable to injuries that disrupt these conductive pathways. While traditional insulating scaffolds fail to bridge this electrophysiological gap, conductive hydrogels have emerged as a transformative paradigm in neural tissue engineering. By mimicking the electrical properties of native tissue, these advanced biomaterials not only reconstruct physical and electrical continuity but also serve as active platforms for localized ES. Ultimately, the synergy between conductive hydrogels, dynamic bioelectric cues, and targeted immunomodulation effectively reshapes the hostile injury microenvironment, substantially enhancing NSC differentiation, axonal regeneration, and comprehensive neural circuit reconstruction.

In summary, endogenous bioelectrical signals act as essential physiological cues across various tissues such as bone, skin, heart, and nerve. Traditional hydrogels, while structurally biomimetic, intrinsically lack electrical conductivity. Consequently, they are unable to respond to endogenous bioelectrical signals or efficiently transmit exogenous electrical stimuli, which substantially restricts their ability to promote tissue regenerative efficacy. To address this, conductive hydrogels have emerged as active physiological regulators that successfully re-establish the local electrophysiological microenvironment, bridge electrically decoupled tissues, and activate intracellular signaling cascades to accelerate tissue repair. Despite these profound advantages, the clinical translation of current conductive hydrogels is severely hindered by the instability of the hydrogel–tissue interface. In physiological environments, the ubiquitous hydration layer acts as a physical barrier that prevents intimate molecular contact between the hydrogel and the underlying tissue. This interfacial separation inevitably leads to mechanical decoupling during dynamic tissue movements and severe signal attenuation due to elevated contact impedance. Therefore, relying solely on electrical conductivity is insufficient. Achieving stable, low-impedance interfacial wet adhesion is a critical prerequisite for high-fidelity bioelectrical signal transmission, fundamentally underscoring the necessity of developing “adhesive-electrocoupling” hydrogels.

## Design of Adhesive-Electrocoupling Hydrogels

### Construction strategy of tissue-adhesive polyphenol hydrogels

#### Challenges of tissue adhesion

**Fig. 6.**
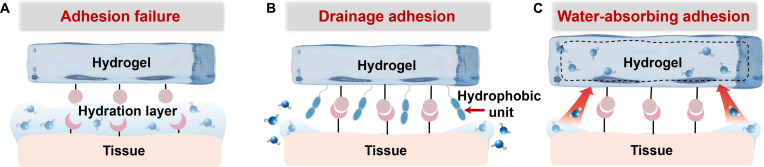
Challenges of tissue adhesion and strategies for removing the hydration layer. (A) Hydration layer on tissue surface hinders adhesion. (B) Hydrogel interfacial drainage promotes tissue adhesion. (C) Hydrogel absorbs hydration layer to promote tissue adhesion.

In the field of hydrogel-based tissue repair, achieving effective adhesion at the tissue interface is a critical prerequisite for ensuring therapeutic efficacy [77]. However, the primary challenge lies in the wettability of the physiological environment. Unlike adhesion mechanisms on dry substrates, in vivo tissue surfaces are consistently covered by a hydrated layer composed of interstitial fluid, blood, lymph, or organ-specific secretions. The presence of this interfacial liquid substantially impedes direct contact between the adhesive groups on the hydrogel surface and the underlying tissue, thereby weakening interfacial adhesion (Fig. 6A). Specifically, water molecules compete with adhesive functional groups for binding sites on the tissue surface, disrupting specific interactions such as hydrogen bonding and electrostatic attraction, and ultimately hindering the formation of a stable adhesive interface. Therefore, effective removal or regulation of the hydrated layer between the hydrogel and tissue is essential to achieving strong wet adhesion.

Currently, 2 proven approaches are employed to address this issue: interfacial drainage and hydration layer absorption [78]. From the perspective of interfacial drainage, most hydrogels easily accumulate water molecules and form a hydration layer due to their hydrophilic surface properties. Thus, regulating the surface wettability of hydrogels is a key strategy to promote interfacial drainage and enhance wet adhesion. Incorporating hydrophobic components by introducing hydrophobic monomers (such as those containing long alkyl chains or aromatic rings) is a common and effective method. These hydrophobic monomers repel water molecules at the hydrogel–tissue interface via hydrophobic interactions, thereby expelling the hydration layer (Fig. 6B). In aqueous environments, the hydrophobic backbones of the hydrogel network, combined with hydrophilic adhesive pendant groups, rapidly self-aggregate to form micelle-like structures that simultaneously repel interfacial water and expose adhesive groups [79]. This synergistic mechanism enables rapid and robust adhesion to substrates in diverse liquid environments, including deionized water, seawater, phosphate-buffered saline, and solutions with a wide pH range (3 to 11). In addition, aromatic functional groups, due to their inherent hydrophobicity, can act as interfacial water drains within the hydrogel matrix, protecting multiple hydrogen bonds between the hydrogel and tissue from water interference and thereby ensuring strong wet adhesion [80].

From the perspective of hydration layer absorption, rapidly absorbing interfacial water can effectively expand the contact area between the hydrogel and tissue, enhancing wet adhesion (Fig. 6C). Post-desolvation treatment of hydrogels with ethanol can modulate polymer chain entanglement, creating a desolvated network capable of rapidly absorbing interfacial water upon contact with wet tissue [81]. This process disrupts the hydration layer, allowing adhesive groups within the hydrogel to directly interact with the tissue surface. Similarly, incorporating highly hygroscopic components into hydrogels can actively absorb interfacial water, breaking down the hydration barrier and establishing a stable adhesive interface. Hygroscopic hydrogel powders can also absorb interfacial liquid and rapidly gel to form a viscous hydrogel layer, achieving effective wet adhesion [82]. When choosing between these 2 strategies, hydrophobic drainage is highly advantageous for dynamic, fluid-rich environments where continuous and active fluid displacement is necessary to prevent adhesion failure. Conversely, hydration layer absorption is often more suitable for relatively static wet tissues or situations where rapid initial adhesion is required, as it relies on the hydrogel network rapidly swelling to absorb interfacial moisture. The choice is also dictated by the polymer matrix design. If the backbone consists of highly hydrophilic and hygroscopic networks, leveraging hydration layer absorption is structurally more favorable. However, if the polymer backbone can be engineered to incorporate hydrophobic domains, hydrophobic drainage becomes the preferred mechanism to repel interfacial water.

It is worth noting that beyond the static challenge posed by the hydration layer, the dynamic nature of biological tissues introduces additional complexity for maintaining long-term adhesion [83]. The physiological environment is mechanically active, and cardiac pulsation, gastrointestinal peristalsis, and muscle contraction constantly deform tissues. Therefore, hydrogel–tissue interfaces must not only exhibit strong initial adhesion but also sustain repeated shear, tensile, and compressive stresses to prevent adhesion failure under dynamic physiological conditions.

#### Mussel-inspired adhesion

**Fig. 7.**
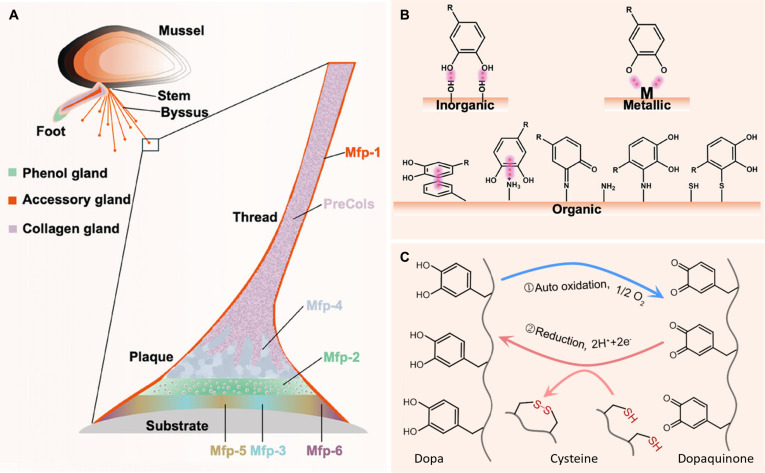
Mussel adhesion mechanism. (A) Mussel and mussel foot proteins. Reproduced with permission from [85]. Copyright 2025, Wiley-VCH. (B) Interaction of DOPA with the surface of the adherent substrate. Reproduced with permission from [85]. Copyright 2025, Wiley-VCH. (C) Redox balance of DOPA. Reproduced with permission from [90]. Copyright 2019, Elsevier.

Biomimetic adhesion mechanisms provide valuable natural models for achieving durable wet adhesion in hydrogels. Among them, the strong underwater adhesion of marine mussels has attracted particular research interest [84]. Mussels can firmly attach to various submerged surfaces—such as rocks and ship hulls—despite constant exposure to water currents. This remarkable capability arises from 6 key mussel foot proteins (Mfps) secreted by the mussel foot: Mfp-1, Mfp-2, Mfp-3, Mfp-4, Mfp-5, and Mfp-6 [85] (Fig. 7A). These proteins rapidly solidify to form adhesive plaques characterized by exceptional interfacial bonding strength, durability, and toughness. The molecular basis of their strong adhesion lies in the high content of DOPA, lysine, and cysteine residues within their sequences [86]. These functional residues are often positioned adjacently along the protein backbone, enabling synergistic interactions that enhance adhesion.

The catechol groups of DOPA serve as core adhesive sites, establishing initial, stable bonds with substrate surfaces through various noncovalent interactions, including hydrogen bonding, cation–π interactions, and π–π stacking. Furthermore, in seawater at approximately pH 8.2, catechol groups readily oxidize to form dopaquinone, which can react with nucleophilic functional groups on the substrate to form covalent linkages, such as Schiff base reactions with amines and Michael addition reactions with thiols, further strengthening interfacial bonding [85] (Fig. 7B). Notably, Mfps-mediated adhesion is not solely dependent on DOPA. Lysine and hydrophobic residues can physically disrupt the hydration layer on substrate surfaces [87,88]. Surface force analyses have confirmed that the amine groups of lysine residues actively displace interfacial water molecules and cooperate with catechol groups to substantially enhance wet adhesion [89].

Furthermore, cysteine-containing Mfps confer precise redox control over the oxidation state of DOPA through dynamic thiol-disulfide exchange reactions. These cysteine residues can reduce oxidized dopaquinone back to DOPA, thereby maintaining the concentration of the adhesive-active form [90] (Fig. 7C). This redox cycling mechanism not only prevents excessive oxidation of DOPA, preserving its adhesive functionality, but also enables real-time repair of interfacial interactions. Through this dynamic regulation, Mfps maintain a robust and durable adhesive interface with the substrate. These insights provide critical molecular-level guidance for the biomimetic design of next-generation adhesive hydrogels.

#### Polyphenol-based adhesive hydrogel

Polyphenols, owing to the presence of catechol and pyrogallol groups in their molecular structures, share structural and functional similarities with DOPA found in Mfps [13]. These catechol groups serve as versatile “molecular anchors”, enabling polyphenols to replicate the robust adhesion of mussels through a repertoire of interactions, including strong hydrogen bonding, metal coordination, cation–π interactions, and oxidative crosslinking [84]. Polyphenols are widely distributed in nature, occurring abundantly in plants and marine organisms. To date, more than 8,000 polyphenolic compounds have been identified, with representative examples including dopamine (DA), tannic acid (TA), gallic acid (GA), and epigallocatechin gallate (EGCG) [91]. Due to their high natural abundance, good biocompatibility, and diverse chemical properties, polyphenols have been widely used in the design, research, and application of mussel-mimicking adhesive materials [92]. In a pioneering study in 2002, Lee et al. [93] modified polyethylene glycol (PEG) with DOPA to synthesize a PEG-DOPA copolymer, which was subsequently crosslinked through oxidative polymerization to form a hydrogel with strong wet adhesion. This work established the first polyphenol-based biomimetic hydrogel system and laid a solid foundation for the further development of mussel-inspired adhesive hydrogels. However, translating these fundamental biomimetic principles into robust practical applications still faces 3 critical challenges: the interference of interfacial hydration layers, the trade-off between adhesion and cohesion, and the chemical instability of catechol groups [11,15,77]. Consequently, recent strategies have focused on overcoming these barriers by manipulating the hydrophilic–hydrophobic balance to remove interfacial water, reinforcing the hydrogel network via oxidative crosslinking, and creating nanoconfined environments to prevent catechol overoxidation.

**Fig. 8.**
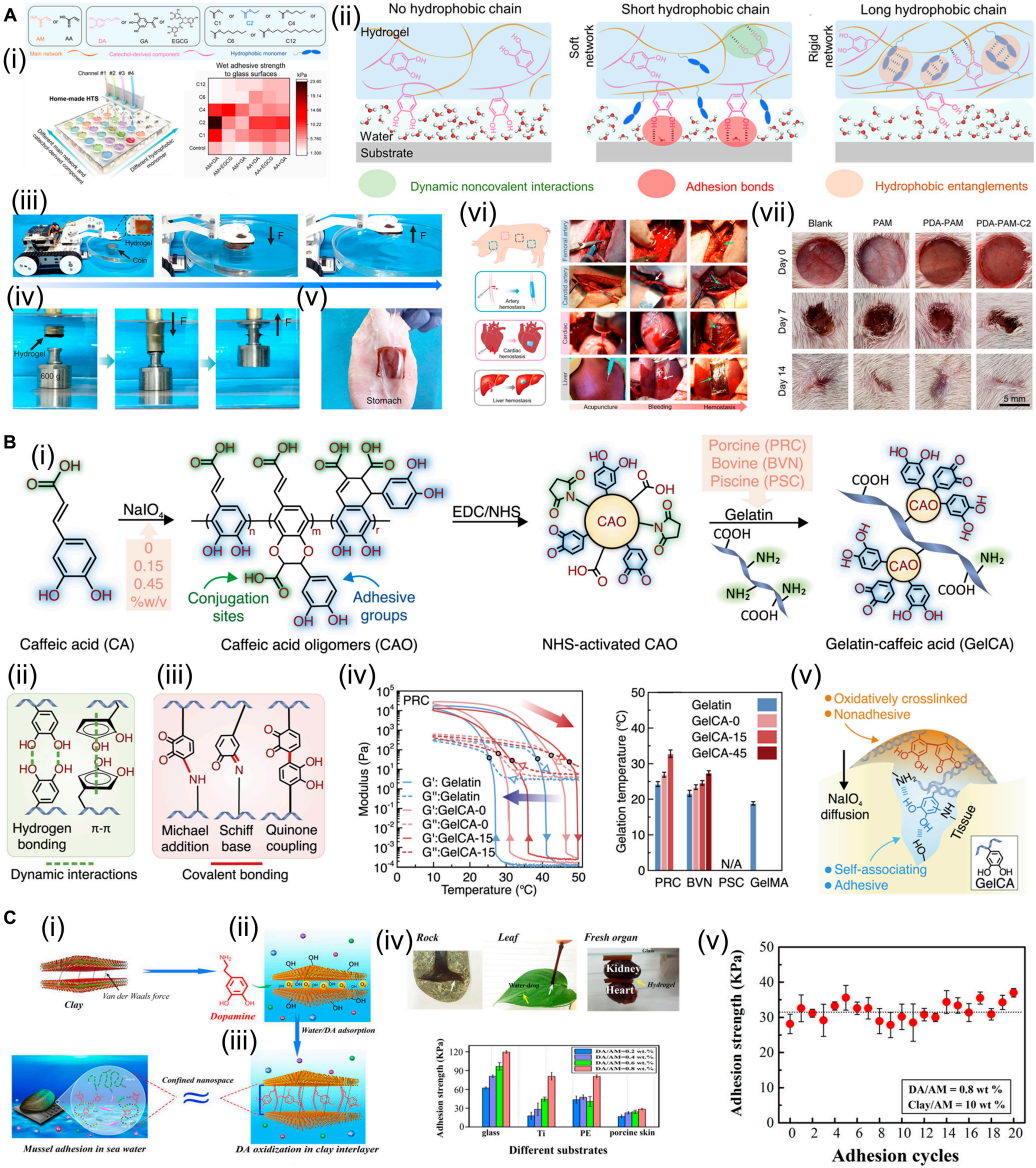
Construction of polyphenol-based adhesive hydrogel. (A) Drainage-adhesive polyphenol hydrogel. Reproduced with permission from [94]. Copyright 2023, American Chemical Society. (i) High-throughput screening of hydrogel components. (ii) Schematic diagram of the wet adhesion mechanism of different hydrogels. (iii) Wet-adhesive hydrogel quickly picks up light objects from water. (iv) Wet-adhesive hydrogel applies pressure to pick up heavy objects from water. (v) Wet-adhesive hydrogels stably adhere to porcine stomach under flushing. (vi) Wet-adhesive hydrogels for hemostasis. (vii) Wet-adhesive hydrogels for wound repair. (B) Polyphenols enhance hydrogel cohesion and achieve wet adhesion. Reproduced with permission from [95]. Copyright 2023, Elsevier. (i) GelCA preparation process. (ii) Noncovalent interactions within hydrogels. (iii) Covalent interactions within hydrogels. (iv) The gel–sol transition temperature increases with the increase of catechol content. (v) One-way adhesion is achieved after the surface catechol groups are consumed. (C) Long-term adhesion of hydrogels by inhibiting polyphenol oxidation via nanoconfined space. Reproduced with permission from [97]. Copyright 2017, American Chemical Society. (i) Nanostructure of clay nanosheets. (ii) Inserting DA into the nanospace of clay nanosheets to form PDA–clay composites. (iii) The interlayers of clay nanosheets create confined nanospaces similar to mussel plaques. (iv) The hydrogel exhibits strong adhesion to various substrates. (v) Adhesion strength of hydrogel during 20 peel-and-stick cycles on porcine skin.

Effective underwater adhesion can be achieved by promoting surface water drainage, regulating the hydrophilic–hydrophobic balance, and introducing catechol groups onto biomaterial surfaces. Hou et al. [94] systematically developed wet-adhesive hydrogels through high-throughput screening of various hydrophobic alkyl monomers and catechol-derived adhesive components (Fig. 8A). Utilizing a custom-built high-throughput screening platform, the authors efficiently and comprehensively evaluated the structure–property relationships among hydrophobic alkyl monomers of different chain lengths, catechol-based adhesive moieties, and hydrophilic polymer networks (Fig. 8A-i). The results revealed that hydrophobic monomers play a dominant role in wet adhesion. Specifically, monomers with appropriately short alkyl chains exhibited the strongest wet adhesion by effectively repelling interfacial water molecules, whereas longer alkyl chains primarily formed strong hydrophobic interactions within the hydrogel matrix, thereby hindering or dissipating the energy required for interfacial bonding (Fig. 8A-ii). The developed wet-adhesive hydrogel can rapidly pick up a lightweight coin from water without the need for strong pressure (Fig. 8A-iii). It can also adhere firmly to a metal block under pressure and stably lift it out of the water (Fig. 8A-iv). Moreover, the hydrogel maintained strong wet adhesion to the porcine stomach even in turbulent water conditions (Fig. 8A-v). Incorporating synergistic catechol and hydrophobic moieties, the engineered wet-adhesive hydrogel exhibits exceptional biocompatibility, substantially enhancing cell affinity and promoting robust cellular adhesion. The resulting wet-adhesive hydrogels demonstrated excellent performance in rapid hemostasis (Fig. 8A-vi) and wound repair (Fig. 8A-vii).

In addition to precisely regulating the hydrophilic–hydrophobic balance of polyphenol-based hydrogels to establish a stable wet adhesion interface, enhancing the intrinsic cohesion of the hydrogel adhesive itself represents another effective strategy for improving wet tissue adhesion. This enhancement in cohesive strength is commonly achieved through oxidative crosslinking reactions of polyphenolic components. Montazerian et al. [95] synthesized caffeic acid oligomers (CAOs) via oxidative polymerization of caffeic acid (CA), producing oligomers with a higher catechol group density than the monomeric CA. The CAO molecules were then covalently conjugated to gelatin through carbodiimide chemistry using 1-ethyl-3-(3-dimethylaminopropyl)carbodiimide (EDC)/*N*-hydroxysuccinimide (NHS), yielding catechol-functionalized gelatin (GelCA) (Fig. 8B-i). When blended with gelatin methacrylate (GelMA) to form a composite hydrogel, the abundant phenolic hydroxyl groups of CAO promoted extensive hydrogen bonding and π–π interactions (Fig. 8B-ii). In addition, Michael addition and Schiff base reactions occurred between primary amine groups in gelatin and the carbonyl groups in CAO (Fig. 8B-iii), enhancing the hydrogel’s internal cohesion and increasing its gel–sol transition temperature, which rose with increased catechol content on GelCA (Fig. 8B-iv). The pregel solution remained injectable and rapidly gelled upon injection into a wound, resisting disintegration in body fluids and maintaining stability at physiological temperature. Subsequent infiltration of the hydrogel with NaIO_4_ generated a gradient crosslinking profile across its thickness. The surface layer became densely crosslinked through catechol coupling reactions, forming a compact, locally crosslinked barrier that prevented hydrogel dissolution under wet physiological conditions. Upon oxidation by NaIO_4_, the surface catechol groups were consumed, producing a nonadhesive outer layer that enabled “one-way adhesion”, in which only the internal surface remained adhesive (Fig. 8B-v). Meanwhile, the hydrogel demonstrates excellent biocompatibility, sustaining in vitro cell viability above 80% and exhibiting no discernible tissue toxicity following a 4-week implantation period in murine models. Similarly, Zhao et al. [96] employed H_2_O_2_/horseradish peroxidase to crosslink hydrogel networks through oxidative coupling between catechol groups and covalent bonding between catechol and amino functionalities. This dual crosslinking strategy imparted the hydrogel with anti-swelling behavior, high mechanical strength, and strong cohesive properties. Moreover, further crosslinking between oxidized catechol groups and amino or thiol groups at tissue interfaces enhanced tissue adhesion under physiological conditions.

The catechol group of DOPA is crucial for the adhesion function of mussels. However, in the alkaline environment of seawater and under continuous exposure to dissolved oxygen, DOPA is prone to oxidation, leading to the loss of active catechol groups [85]. This overoxidation substantially reduces catechol content and thereby weakens or even abolishes adhesive performance. To overcome this challenge, mussels have evolved protective mechanisms that prevent the overoxidation of DOPA within their Mfps, ensuring sustained adhesion under marine conditions. Inspired by this biological strategy, preventing the overoxidation of catechol groups has become a crucial approach for developing long-term, reusable, polyphenol-based adhesive hydrogels. The most straightforward way to prevent catechol overoxidation is to reduce their exposure to oxygen. Han et al. [97] constructed a nanoconfinement space to prevent excessive oxidation of PDA in a poly(acrylamide) (PAM) hydrogel matrix, thereby achieving long-term adhesion (Fig. 8C). The nanoconfined space is formed by clay nanosheets with a layered structure (Fig. 8C-i). After embedding DA molecules between clay nanosheets, the Mg^2+^, Ca^2+^, and Na^+^ ions released from the clay created an alkaline microenvironment similar to seawater, allowing DA to undergo in situ oxidative polymerization within the confined nanospaces between the clay layers, forming PDA and yielding PDA–clay composites (Fig. 8C-ii). The limited oxygen diffusion and spatial confinement effectively restricted excessive oxidation, preserving a high concentration of active catechol groups within the PDA (Fig. 8C-iii). Subsequently, acrylamide monomers, crosslinkers, and initiators were added to the PDA–clay dispersion to form a PDA–clay–PAM hydrogel. Benefiting from the nanoconfinement effect of the intercalated PDA–clay structure, the resulting hydrogel mimics the adhesion mechanism of mussels by retaining abundant free catechol groups and demonstrating strong adhesion to a wide range of substrates (Fig. 8C-iv). It also shows reproducible and long-lasting adhesion to porcine skin (Fig. 8C-v). This mussel-inspired PDA–clay–PAM hydrogel exhibits excellent biocompatibility, demonstrating profound cellular affinity during in vitro evaluations. It substantially promotes the adhesion, spreading, and proliferation of fibroblasts. Furthermore, the hydrogel demonstrated exceptional in vivo safety within a rat full-thickness skin defect model, effectively mitigating inflammation while markedly accelerating tissue regeneration and wound closure.

### Polyphenol-mediated adhesive-electrocoupling hydrogels

#### Conductive hydrogels

Bioelectricity, the fundamental energy and information carrier of living organisms, is deeply involved in maintaining tissue homeostasis, regulating cell fate, and promoting regeneration after injury [98]. It plays indispensable roles in guiding immune cell migration during inflammatory regulation and directing cell differentiation for tissue repair [99]. However, traditional hydrogels lack electrical conductivity. As a result, they cannot respond to endogenous bioelectrical signals within the body for dynamic physiological monitoring, nor can they effectively transmit therapeutic cues through exogenous ES to facilitate functional tissue recovery. These shortcomings substantially restrict the potential of traditional hydrogels in tissue engineering and regenerative medicine. Therefore, to fully harness the regulatory functions of bioelectricity, it is crucial to endow hydrogels with electrical conductivity.

**Fig. 9.**
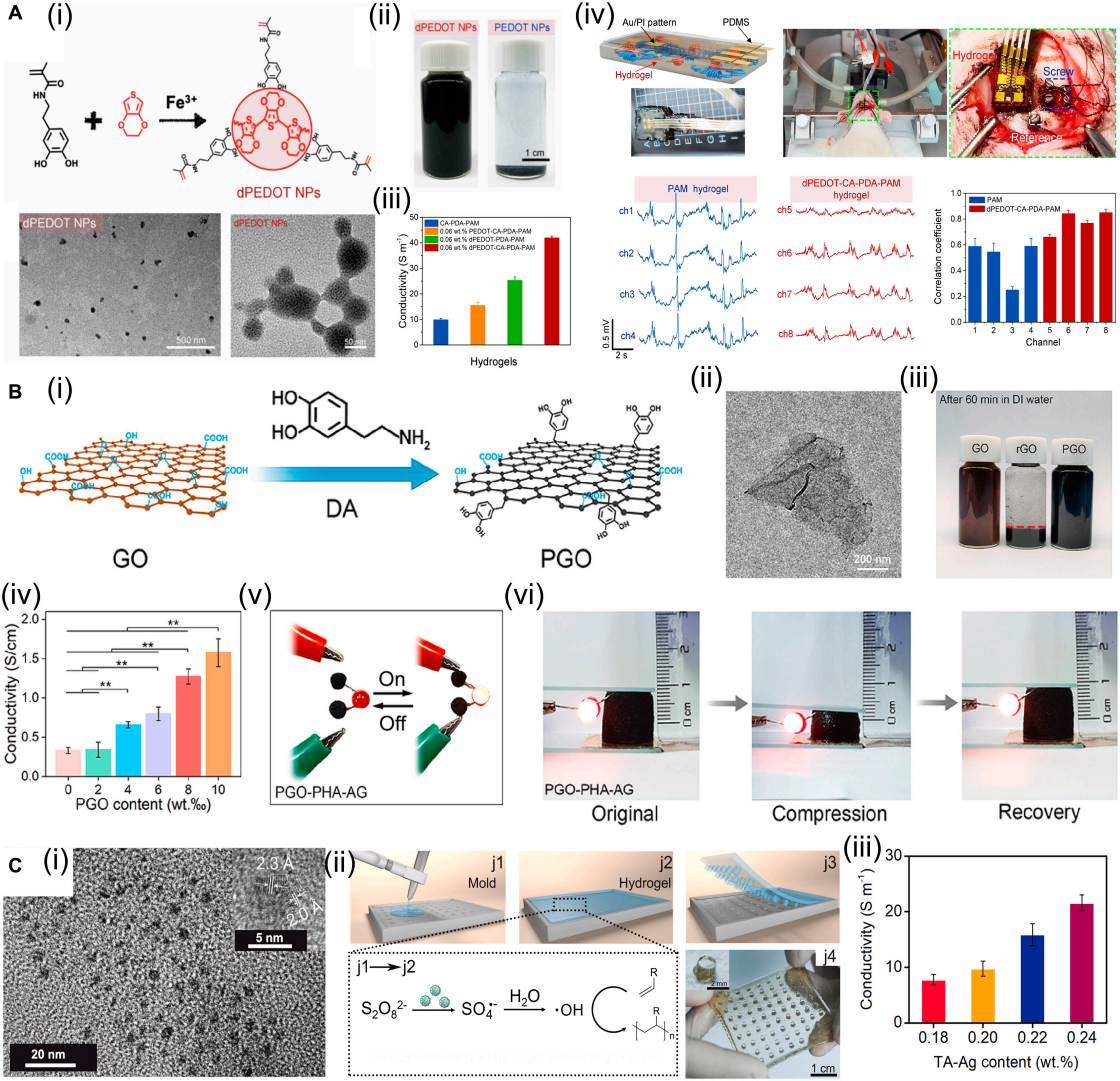
Construction of conductive hydrogels. (A) Conductive hydrogel based on conductive polymer. Reproduced with permission from [103]. Copyright 2022, Elsevier. (i) Schematic diagram of dPEDOT NP synthesis and its morphology. (ii) Water dispersibility of dPEDOT NPs. (iii) Conductivity of hydrogel. (iv) Hydrogels for brain–computer interfaces. (B) Conductive hydrogel based on carbon nanomaterial. Reproduced with permission from [105]. Copyright 2022, Elsevier. (i) Schematic diagram of PGO synthesis. (ii) Transmission electron microscopy (TEM) image of PGO. (iii) Digital images of GO, reduced GO, and PGO after dispersion in water for 60 min. (iv) Electrical conductivity of scaffolds with different PGO contents. (v) The LED is lit in the circuit connected in series with the scaffold. (C) Conductive hydrogel based on metal NPs. Reproduced with permission from [106]. Copyright 2021, Elsevier. (i) TEM image of TA–Ag nanozymes. (ii) TA–Ag nanozymes catalyze hydrogel formation. (iii) Conductivity of hydrogels with different TA–Ag nanozyme contents.

Incorporating conductive fillers such as conductive polymers, carbon-based nanomaterials, and metal nanomaterials into hydrogel matrices is an effective strategy for constructing conductive hydrogels and has been extensively investigated [100,101]. Zhang et al. [102] successfully grafted poly(pyrrole) (PPy) onto nonconductive gelatin molecules to form a gelatin-PPy (GP) hydrogel through a Schiff-base reaction with oxidized xanthan gum. GP not only improved the water dispersibility of the conductive polymer but also provided the hydrogel with ideal conductivity. This injectable conductive hydrogel exhibits mechanical and electrical properties that closely match those of native myocardial tissue, alongside excellent biocompatibility. Post-implantation, it safely promotes myocardial repair while effectively mitigating inflammation. Wang et al. [103] developed hydrophilic DA methacrylate-hybridized poly(3,4-ethylenedioxythiophene) nanoparticles (dPEDOT NPs) (Fig. 9A-i). When incorporated into a carrageenan-interpenetrating PEDOT-PAM hydrogel, the excellent hydrophilicity of dPEDOT NPs enabled their stable and uniform dispersion within the hydrogel network (Fig. 9A-ii), forming efficient conductive pathways and improving the conductivity of the hydrogel (Fig. 9A-iii). The resulting hydrogel exhibited outstanding bioelectronic properties and was successfully applied in brain–computer interfaces for precise and long-term electroencephalographic (EEG) signal acquisition (Fig. 9A-iv). Demonstrating high mechanical compatibility with brain tissue, this ultra-soft conductive hydrogel possesses remarkable immune evasion capabilities. It effectively suppresses foreign body reactions and neuroinflammation, thereby exhibiting exceptional in vivo biocompatibility.

Carbon nanomaterials have also been widely employed to construct conductive hydrogels. Han et al. [104] designed and synthesized PDA-modified carbon nanotubes (PDA-CNTs), which, owing to the excellent hydrophilicity of PDA, could be uniformly dispersed in aqueous media. When introduced as functional fillers into a glycerol/water binary solvent hydrogel, PDA-CNTs endowed the composite with frost resistance, heat resistance, and long-term stability. The superior dispersibility of PDA-CNTs ensured their homogeneous distribution within the gel matrix, thereby forming a robust conductive network that maintained stable conductivity across a wide temperature range (−20 to 60 °C). Demonstrating superior biocompatibility and robust tissue adhesion, this mussel-inspired hydrogel can be safely applied to human skin for the detection of physiological signals. Additionally, it functions as an effective dressing to shield the skin against thermal injuries, such as frostbite and burns. Li et al. [105] leveraging the high redox activity and strong binding capacity of PDA, further reduced graphene oxide (GO) to PDA-functionalized graphene (PGO) (Fig. 9B-i), which exhibited a characteristic wrinkled sheet-like morphology (Fig. 9B-ii). Compared with GO reduced by iodic acid, PGO demonstrated markedly improved water dispersibility (Fig. 9B-iii). Incorporating PGO as a conductive filler into alginate/gelatin scaffolds creates efficient conductive pathways (Fig. 9B-iv) and enhances the electrical conductivity of the scaffolds (Fig. 9B-v). Moreover, it can maintain stable conductivity even under mechanical deformation (Fig. 9B-vi). Equipped with immunomodulatory functions, including the scavenging of ROS and anti-inflammatory properties, this conductive scaffold demonstrates excellent tissue compatibility. It safely and efficaciously promotes periodontal bone regeneration, even within diabetic microenvironments.

Metals are inherently excellent electrical conductors, and nanoscale forms of metal nanomaterials not only retain this high conductivity but also exhibit remarkable chemical stability. Among them, silver (Ag) is one of the most widely utilized metals in biomaterials, with several Ag-based products already approved by the FDA. Jia et al. [106] using the natural polyphenol TA and ultrasmall Ag NPs as precursors, successfully synthesized ultrasmall TA–Ag conductive nanozymes through chelation, achieving an average particle diameter of approximately 5 nm (Fig. 9C-i). These TA–Ag nanozymes preserve abundant surface phenolic hydroxyl groups, a key structural feature that facilitates uniform dispersion within hydrogel matrices. In addition, the nanozymes display excellent catalytic activity, enabling spontaneous hydrogel formation without the need for external stimuli (Fig. 9C-ii). Their homogeneous distribution within the hydrogel network further endows the composite material with superior electrical conductivity (Fig. 9C-iii), thereby broadening the functional applications of conductive hydrogels in bioelectronic and regenerative systems. Featuring a highly hydrated structure analogous to native soft tissue, this mussel-inspired hydrogel exhibits superior biocompatibility. Consequently, it can be safely utilized both as a wound dressing to facilitate tissue regeneration and as a material for implantable devices. In addition to Ag, gold (Au) NPs and nanorods are also widely employed for constructing conductive hydrogels based on metallic nanomaterials. Xu et al. [107] successfully developed a conductive hydrogel using O-carboxymethyl chitosan (CMC), an aldehyde-terminated polyurethane (DAPU) nanocrosslinker, and Au NPs as key components. In this system, the Au NPs not only impart electrical conductivity to the hydrogel but also promote the exposure of aldehyde groups on DAPU, which subsequently undergo a Schiff-base reaction with the amino groups of CMC, thereby facilitating hydrogel formation. This biodegradable conductive hydrogel exhibits robust anti-inflammatory properties and supports the proliferation and differentiation of NSCs. Furthermore, it demonstrates favorable neural tissue compatibility and biosafety following in vivo injection. Brain injection of the hydrogel rescues motor function in Parkinson’s disease rats.

#### Adhesive-electrocoupling hydrogels

**Fig. 10.**
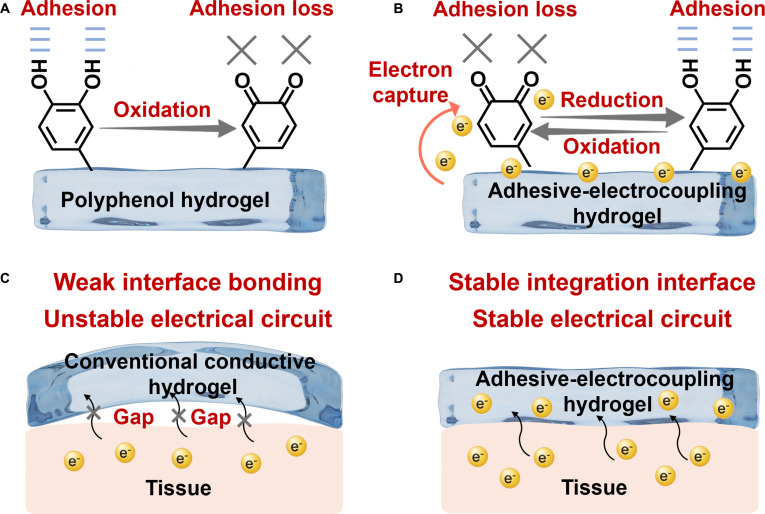
Adhesive-electrocoupling hydrogel. (A) Polyphenols lose their adhesion after oxidation. (B) Adhesive-electrocoupling hydrogel maintains adhesion by maintaining catechol content in polyphenol hydrogel through electron transfer. (C) Weak interfacial bonding leads to unstable circuits at the hydrogel–tissue interface. (D) Adhesive-electrocoupling hydrogel promotes long-term adhesion and stable electrical signal transmission.

Although polyphenols have a structure similar to DOPA, making them ideal biomimetic adhesive units, their susceptibility to oxidation causes a rapid loss of adhesion and insufficient long-term adhesive performance, ultimately leading to interface integration failure (Fig. 10A). In nature, the phenol–quinone conversion is a fundamental mechanism for long-term adhesion in mussels. The key to sustaining a sufficient supply of available catechol groups lies in the continuous secretion of antioxidant proteins, which reduce dopaquinone to dopa, establishing a dynamic redox system [108]. However, the traditional approach of adding reducing agents to hydrogels has substantial drawbacks. Firstly, many reducing agents lack biocompatibility, potentially causing side effects or complications. Secondly, the addition process is often cumbersome and complex, making it unsuitable for implantable adhesive hydrogels.

Redox reactions, essentially electron transfer processes, present a more elegant solution [11]. Our previous research, through quantum mechanical calculations, uncovered the electron transfer mechanism between polyphenols, metals, and conductive polymers [106]. Conductive materials can transfer electrons to quinone groups, reducing them back to phenolic hydroxyl groups, thus preserving the catechol content in the hydrogel and ensuring long-term adhesion (Fig. 10B). Conventional conductive hydrogels lack intrinsic adhesion, leading to weak interfacial bonding, the formation of gaps between the hydrogel and wet tissue, and unstable electrical circuits at the hydrogel–tissue interface (Fig. 10C). By utilizing the relationship between dynamic adhesion and electron transfer of mussels, bioelectric signal conduction and phenolquinone conversion of polyphenols are effectively coupled to construct an adhesive-electrocoupling hydrogel [58]. In this way, conductive materials not only provide electrical conductivity but also maintain the catechol group content via electron transfer, promoting sustained adhesion. This long-term adhesion, in turn, stabilizes conductivity, supports prolonged transmission of bioelectrical signals (Fig. 10D).

**Fig. 11.**
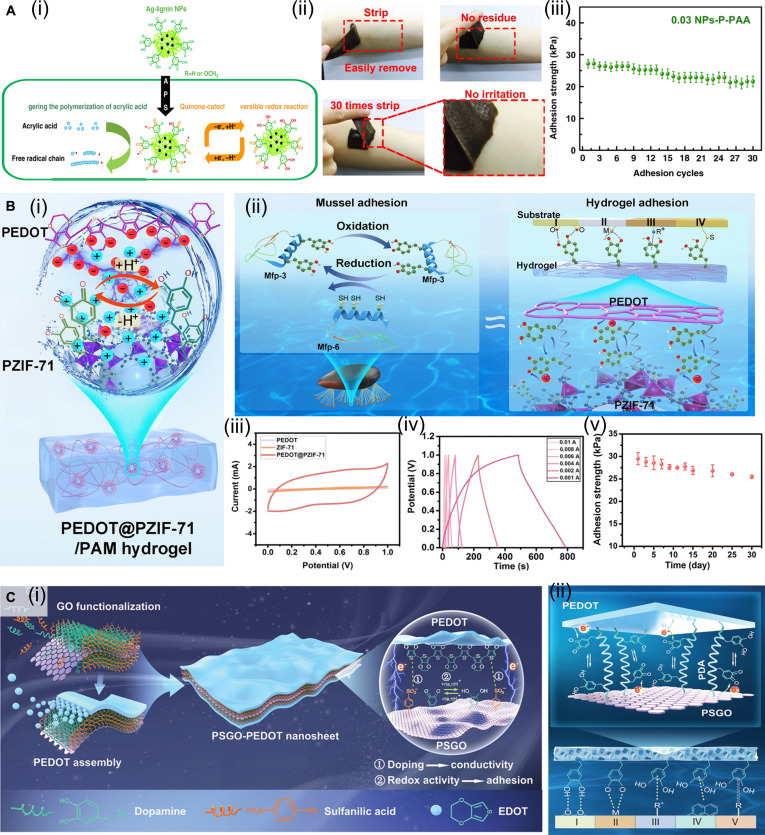
Construction strategy of adhesive-electrocoupling hydrogel. (A) Adhesive-electrocoupling hydrogel based on redox reaction of Ag–lignin system. Reproduced with permission from [109]. Copyright 2019, Springer Nature. (i) Reversible redox reaction of quinone–catechol in the Ag–lignin system. (ii) Continuous and reproducible adhesion of hydrogel. (iii) Adhesion strength of hydrogel during 30 peel-and-stick cycles on porcine skin. (B) Adhesive-electrocoupling hydrogel based on redox reaction of PEDOT@PZIF-71 system. Reproduced with permission from [111]. Copyright 2023, Royal Society of Chemistry. (i) Redox reactions in PEDOT@PZIF-71. (ii) Electron transfer in confined nanospaces promotes phenol–quinone conversion to achieve mussel-like adhesion. (iii) Cyclic voltammetry curves of PEDOT@PZIF-71. (iv) Galvanostatic charge–discharge curves of PEDOT@PZIF-71 at different currents. (v) Adhesion strength of hydrogel during storage at 4 °C for 30 d on porcine skin. (C) Adhesive-electrocoupling hydrogel based on redox reaction of PSGO-PEDOT nanosheets. Reproduced with permission from [112]. Copyright 2019, Wiley-VCH. (i) Schematic diagram of the preparation of PSGO-PEDOT nanosheets. (ii) Reversible redox conversion of phenolic quinone via electron transfer between PSGO and PEDOT layers.

Inspired by the redox mechanism of mussels, Gan et al. [109] proposed an innovative strategy for constructing hydrogels with long-lasting adhesive properties, centered on a dynamic redox system triggered by Ag–lignin NPs (Fig. 11A). Lignin, a plant-derived biopolymer, contains abundant phenolic hydroxyl groups within its molecular structure. These phenolic hydroxyl groups can reduce Ag^+^ ions to form Ag–lignin NPs, during which they are oxidized to the corresponding quinone groups. As Ag NPs are typical plasmonic metals, the surface plasmon resonance effect of the Ag–lignin NPs transfers free electrons to the quinone groups of lignin, thereby converting them back into catechol groups (Fig. 11A-i). This process mimics the adhesion mechanism of mussel foot proteins, maintaining a dynamic redox balance and continuously supplying reactive catechol groups required for adhesion, thereby enabling sustained and repeatable adhesion of the hydrogel (Fig. 11A-ii). As a result, the hydrogels exhibit durable and reproducible adhesion. The adhesive strength remained stable after 30 peel-and-stick cycles (Fig. 11A-iii). Exhibiting excellent cellular affinity and potent antibacterial activity, this adhesive-electrocoupling hydrogel demonstrates superior biocompatibility. Consequently, it serves as an ideal candidate for soft tissue repair and wound closure. Overall, this study demonstrates that electron transfer provides an effective mechanism for establishing a dynamic catechol/quinone redox equilibrium in self-adhesive bioelectronic hydrogels. In addition, conductive polymers exhibit excellent electrical conductivity and can also form electron donor–acceptor complexes with catechol-based materials, thereby facilitating electron transfer between catechol and quinone groups during the construction of conductive hydrogels and preventing adhesion failure caused by excessive oxidation of catechol groups [110].

The hydrogels prepared by the dynamic redox strategy exhibited excellent electroactivity, and long-term, reproducible adhesion, highlighting their potential in tissue regeneration and ES for implantable bioelectronics. Building upon this concept, a subsequent study designed a mussel-inspired core-shell redox-active system consisting of a PDA-modified zeolitic imidazolate framework (ZIF-71) core and a PEDOT shell [111]. Owing to PDA’s abundant catechol groups, PEDOT can assemble on the surface of ZIF-71, forming a redox-active composite system (Fig. 11B-i). The PDA and PEDOT components form an electron donor–acceptor complex, initiating redox reactions between catechol and quinone groups within the PDA layer. Meanwhile, the PEDOT shell enhances electron transfer efficiency and accelerates redox cycling within the confined nanocapsule space (Fig. 11B-ii). This synergistic amplification of redox dynamics substantially improves the electrochemical performance of the PEDOT@PZIF-71 NPs (Fig. 11B-iii, iv), making them ideal redox-active nanofillers for constructing adhesive and electroactive PAM hydrogels with energy-storage capabilities. The resulting PEDOT@PZIF-71/PAM hydrogel exhibited strong adhesion to a variety of substrates with reproducible long-term adhesion, maintaining strong adhesion to porcine skin after 30 peel-and-stick cycles (Fig. 11B-v). These hydrogels can intimately conform to tissue surfaces, enabling reliable physiological signal detection and stable electrochemical performance. Furthermore, it exhibits excellent in vitro cytocompatibility and negligible cytotoxicity. Upon direct in vivo implantation for continuous bioelectrical monitoring, the material elicited no discernible inflammatory response, thereby confirming its exceptional immunocompatibility and overall biosafety.

Constructing confined nanostructures with charged layers is also an effective strategy for facilitating the redox reactions of phenol–quinone groups. Gan et al. [112] assembled PEDOT onto PDA-grafted and sulfonated GO (PSGO) to form redox-active PSGO-PEDOT nanosheets (Fig. 11C). First, sulfonate groups were introduced onto the surface of GO to obtain sulfonated GO (SGO). Next, catechol groups were incorporated into SGO through PDA functionalization to yield PSGO. The sulfonate groups on PSGO served as doping sites for PEDOT, while the catechol groups enhanced the interaction between PEDOT and PSGO, resulting in electrically conductive PSGO-PEDOT nanosheets with a sandwich-like structure (Fig. 11C-i). PDA and PEDOT formed an electron donor–acceptor complex, generating a confined nanostructured charge layer between the PSGO and PEDOT layers. This structure enhanced carrier mobility and charge transfer from PEDOT and PSGO to the quinone/catechol groups, thereby maintaining the dynamic and reversible redox conversion of these groups in PDA and preserving the abundance of catechol functionalities (Fig. 11C-ii). This adhesive-electrocoupling hydrogel exhibits high in vitro cytocompatibility. When utilized as an implantable bioelectronic device for in vivo biosignal detection, it demonstrates reliable tissue adhesion alongside outstanding in vivo biocompatibility.

By integrating conductive materials and catechol groups through an electron donor–acceptor complex system, reversible interconversion between phenol and quinone states is effectively promoted through electron transfer. This dynamic redox process enhances adhesive electrocoupling, ensuring long-term adhesion and stable conductivity. In summary, by rationally engineering the aforementioned polyphenol-mediated networks and conductive fillers, researchers can unlock the immense potential of these adhesive-electrocoupling hydrogels across diverse tissue repair applications. Depending on their specific structural configurations, these materials can be highly tailored for the repair of bioelectrically sensitive tissues, including bone, skin, myocardium, and nerves. Alternatively, they can be optimized for the rapid sealing and healing of dynamic, fluid-rich environments, including bleeding wounds and gastric perforations. The development of such adhesive-electrocoupling hydrogels represents an innovative strategy for advancing biomedical technologies and lays a strong foundation for future applications in smart tissue repair materials. However, the successful translation of these structural designs into actual tissue regeneration is not merely a macroscopic physical patching process. Instead, it is fundamentally dictated by how these electroactive networks interact with the biological microenvironment at the cellular and molecular levels. The following section systematically elucidates the biological impact of adhesive electrocoupling. It details the specific roles of polyphenols and explores how adhesion combined with bioelectric integration actively regulates cellular behavior to initiate tissue repair cascades.

## Biological Effects of Adhesive Electrocoupling

### Biological effects of polyphenols

From a structural perspective, polyphenols are defined by the abundance of phenolic hydroxyl moieties within their architecture. This specific molecular configuration is critical, as it endows them with exceptional reducing power and high chemical reactivity. Physiologically, such reactivity translates into multidimensional biological utility [113]. Specifically, polyphenols have been shown to play pivotal roles not only in anti-inflammatory, anti-oxidative, and antibacterial defenses but also in the fine-tuning of mitochondrial metabolic function. Given these outstanding properties, polyphenols, as a highly promising class of functional materials, demonstrate broad application prospects in the biomedical field.

#### Anti-inflammatory properties of polyphenols

**Fig. 12.**
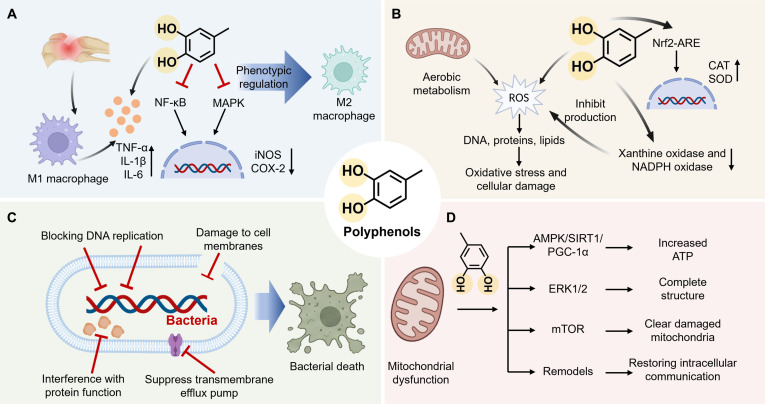
Biological effects of polyphenols. (A) Anti-inflammatory properties of polyphenols. (B) Anti-oxidative properties of polyphenols. (C) Antibacterial properties of polyphenols. (D) Mitochondrial regulatory properties of polyphenols.

As the central coordinator of tissue repair, the immune system precisely regulates the dynamic balance between inflammatory responses, tissue regeneration, and remodeling. Despite inflammation serving as a defensive physiological mechanism to eliminate pathogens, dysregulation of this function or persistent abnormal stimulation can lead to the overactivation of immune effector cells. This disruption of immune homeostasis creates a highly inflammatory microenvironment, which remains a critical barrier to normal tissue repair and acts as a key factor in driving pathological processes [114]. Polyphenolic compounds, with their intrinsic immunomodulatory properties, can directly intervene in these pathways to maintain immune homeostasis without the need for auxiliary agents [115] (Fig. 12A)

For example, at the cellular level, polyphenols can reprogram the spatiotemporal balance of macrophage polarization [116]. While pro-inflammatory M1 macrophages are crucial for initial clearance, an arrested transition to the anti-inflammatory M2 phenotype results in persistent inflammation. Polyphenols facilitate the M1-to-M2 transition, thereby inhibiting the release of inflammatory mediators. Polyphenols have also been shown to substantially inhibit the transcription and secretion of key cytokines such as tumor necrosis factor-α (TNF-α), interleukin-1β (IL-1β), IL-6, and IL-10 in both in vitro and in vivo models [117,118]. Moreover, molecular structure influences targeting specificity and signaling intervention. Specifically, polyphenols can block classical nuclear factor κB (NF-κB) and mitogen-activated protein kinase (MAPK) pathways to down-regulate inducible nitric oxide synthase (iNOS) and cyclooxygenase-2 (COX-2) expression [119]. Variations in chemical composition further dictate function: Flavonoids, like quercetin, inhibit the Janus kinase (JAK)–signal transducer and activator of transcription (STAT) pathway to regulate immune responses, while tannins reshape the immune microenvironment by activating the PI3K/Akt pathway [120,121]. In summary, polyphenols, by constructing a complex regulatory network with multiple targets and pathways, demonstrate powerful anti-inflammatory and immunomodulatory activities.

#### Anti-oxidative properties of polyphenols

Originating from cellular aerobic metabolism, ROS represent a class of highly reactive molecular clusters that includes hydroxyl radicals, superoxide anion radicals, singlet oxygen, and hydrogen peroxide. Despite the efficacy of the body’s sophisticated antioxidant defense system in maintaining physiological homeostasis, dysregulation of this balance occurs when the rate of ROS generation outpaces scavenging capacity. The resulting oxidative stress remains a critical factor in driving pathological processes because excessive ROS nonspecifically modify biomolecules, including DNA, proteins, and lipids, which damages cellular structural integrity and impairs physiological functions.

Polyphenols, with their intrinsic redox regulatory capabilities, can directly influence these oxidative states through their unique molecular architecture, without requiring complex synthetic modifications [113] (Fig. 12B). For example, their structure is characterized by a highly conjugated π–electron system and abundant phenolic hydroxyl groups, a configuration that endows them with exceptional hydrogen atom or electron donor capabilities [122]. This allows polyphenols to rapidly quench free radicals, thereby terminating chain reactions and effectively blocking the cascade of oxidative damage. Polyphenols have also been shown to substantially inhibit the activity of key pro-oxidative enzymes, such as xanthine oxidase and NADPH (reduced form of nicotinamide adenine dinucleotide phosphate) oxidase, curbing the generation of endogenous ROS at its source [123,124]. Moreover, specific coordination sites within their structure facilitate the chelation of redox-active transition metal ions (such as Fe^2+^ and Cu^2+^ to block the metal-catalyzed Fenton reaction, while their ability to act as signal regulators activates the Nrf2-antioxidant response element (ARE) pathway and up-regulates endogenous enzymes like superoxide dismutase and catalase, comprehensively enhancing the cell’s own antioxidant defense [125,126].

#### Antibacterial properties of polyphenols

In recent decades, the global public health crisis caused by bacterial infections has gained substantial attention, particularly regarding the spread of multidrug-resistant bacteria. Despite the historical utility of traditional antibiotics, the evolutionary selection of resistance driven by their overuse remains a critical barrier to reducing clinical mortality and healthcare burdens [127]. Polyphenols, with their intrinsic defensive properties derived from plant secondary metabolism, can directly exert broad-spectrum antibacterial effects through their unique chemical skeletons without requiring single-target mechanisms that favor resistance accumulation [128] (Fig. 12C).

For example, structure–activity relationship studies indicate that hydroxylation modification at the C-5 and C-7 positions of the A ring in flavonoids acts as a key pharmacophore feature [129]. Specific to genetic interference, quercetin and apigenin can precisely intercept replication machinery by competitively interacting with the adenosine triphosphate (ATP)-binding site of the DNA gyrase GyrB subunit, thereby blocking energy supply and inducing DNA strand breaks [130–132]. Catechins have also been shown to disrupt bacterial cell membranes by binding to the lipid bilayer, which inactivates intracellular and extracellular enzymes [133]. Moreover, polyphenols interfere with bacterial protein function via covalent or noncovalent interactions to block nutrient pathways, while specific compounds exhibit unique targeting [134]. Quercetin limits Fe^3+^ uptake required for *Pseudomonas aeruginosa* biofilm formation by up-regulating siderophore proteins, and catechins along with EGCG substantially inhibit transmembrane efflux pumps to restore antibiotic sensitivity in drug-resistant bacteria [135,136].

#### Mitochondrial regulatory properties of polyphenols

As the central hub of cellular energy metabolism and signal transduction, mitochondria play an indispensable role in maintaining cellular homeostasis. Mitochondrial dysfunction is widely recognized as a core pathophysiological link in many complex human diseases, including neurodegenerative diseases and metabolic syndromes, which remains a critical barrier to physiological health [137]. Consequently, targeted mitochondrial regulation has gained substantial attention as a highly promising therapeutic strategy to address these pathological challenges. Polyphenolic compounds, with their intrinsic bioactive properties, have attracted substantial attention due to their ability to exert multidimensional and precise regulation on mitochondrial function, ranging from biogenesis and dynamic balance to overall homeostasis (Fig. 12D).

Variations in chemical structure dictate these diverse regulatory mechanisms through targeted signaling cascade pathways. Specifically, EGCG, a core active ingredient in tea, can effectively restore mitochondrial function by activating the AMPK/SIRT1/PGC-1α signaling axis, thereby promoting AMPK-mediated energy homeostasis remodeling to increase ATP levels for cell differentiation [138]. Similarly, resveratrol has been shown to activate the extracellular signal-regulated kinase 1/2 (ERK1/2) pathway to promote mitochondrial fusion, which maintains the structural integrity of the mitochondrial network [139]. Moreover, regarding mitochondrial homeostasis, curcumin primarily induces mitophagy by regulating the mammalian target of rapamycin (mTOR) signaling pathway to mediate the specific clearance of damaged mitochondria [140]. Furthermore, these regulatory effects extend to sub-organelle interactions. For instance, GA can remodel the mitochondrial–endoplasmic reticulum interface and restore intracellular communication homeostasis by inhibiting the excessive formation of pathological mitochondrial-associated membranes [141].

### Regulating cell behavior using endogenous electrical signals

The precise delivery of electrophysical signals to cells via conductive biomaterials has emerged as a cutting-edge strategy for directing cell fate. Elucidating this interaction requires a fundamental understanding of the charge transport dynamics preceding the cell–material interface [142]. Charge transport typically manifests in unipolar or bipolar modes. The former relies on a single charge carrier type, whereas the latter involves both electrons and holes, often modulated by specific bias or doping conditions. In the realm of flexible biomaterials, conductive hydrogels have garnered substantial attention due to their unique mixed ionic–electronic conductivity. This conductivity arises from the synergistic interaction between a dispersed conductive phase (metals, carbon-based materials, or conductive polymers) and free ions within the aqueous phase. Although metals exhibit superior electronic conductivity, they primarily rely on non-Faradaic processes to form an electrical double layer, which impedes direct charge transfer across the interface into the electrolyte [143,144]. Consequently, this limitation promotes localized charge accumulation, potentially inducing electrochemical damage to cell membranes [142]. In contrast, despite the high defect density of carbon-based materials, their inherent percolation networks allow electrons to bypass defects via tunneling, thereby maintaining macroscopic conductivity [142,145]. Conductive polymers are of particular interest due to their unique bipolar transport capabilities, which enable the conduction of both electrons and holes depending on the material’s redox state [142,146]. Chemical or electrochemical doping, characterized by the introduction of counterions into the conjugated polymer backbone, generates charge carriers such as polarons and bipolarons [147]. This distinct electronic structure enables conductive polymers to efficiently transduce ion–electron signals under physiological conditions, rendering them an ideal interface between abiotic electronic devices and biological systems.

**Fig. 13.**
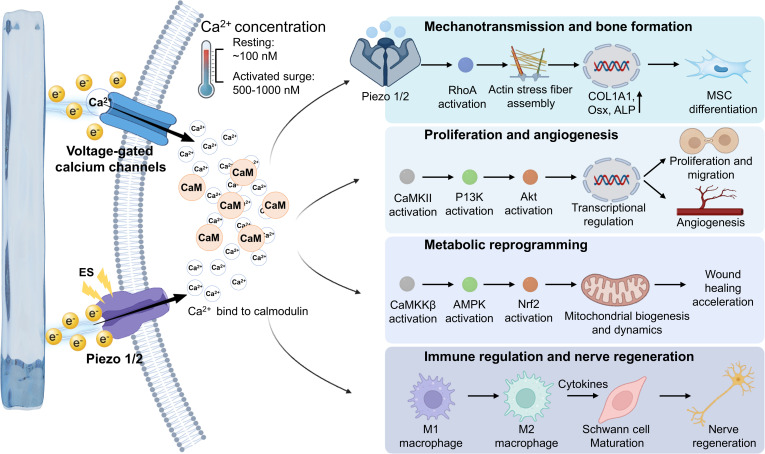
Regulating cell behavior using endogenous electrical signals.

Bioelectricity acts as a fundamental physiological cue within living systems. This phenomenon manifests through endogenous ion fluxes, the spatiotemporal distribution of transmembrane or TEP gradients, and localized electric fields. This intricate electrophysiological microenvironment orchestrates complex cellular behaviors, including morphogenesis, tissue regeneration, cell differentiation, proliferation, and directed migration [142,148]. Cells exhibit profound sensitivity to these bioelectrical cues. Generally, actively proliferating stem or progenitor cells exhibit depolarized membrane potentials, whereas mature differentiated cells maintain a hyperpolarized state [149]. Conductive biomaterials modulate this transmembrane resting potential by regulating charge transfer efficiency at the cell–material interface, effectively mimicking or reshaping cellular electrophysiological states [150]. The central mechanism governing this transduction from physical to biochemical signals lies in the dynamic regulation of intracellular Ca^2+^ [151]. In the resting state, intracellular free Ca^2+^ levels are strictly maintained at a baseline of approximately 100 nM to preserve cellular homeostasis. Upon activation by external ES, however, intracellular Ca^2+^ concentrations can surge to between 500 and 1,000 nM [152]. This elevated calcium influx facilitates binding to effector proteins, such as calmodulin, subsequently triggering downstream enzymatic cascades. Consequently, conductive materials can precisely initiate this electro-calcium signal transduction by controlling nanoscale charge injection, fundamentally reshaping cell–cell communication and cell–matrix interactions (Fig. 13).

Minor perturbations in membrane potential suffice to activate voltage-gated calcium channels (VGCCs) [153]. As primary transducers linking electrophysical signals with intracellular biochemical reactions, these channels play a decisive role in determining stem cell fate. Specifically, regarding mechanoelectric coupling, ES acts directly on ion channels while simultaneously enhancing cellular mechanosensitivity [154]. Under in situ ES generated by piezoelectric materials, the expression of the mechanosensitive ion channel Piezo2 is substantially up-regulated. The resulting Piezo2-mediated calcium influx activates RhoA and its downstream effectors, thereby controlling actin stress fiber assembly and focal adhesion maturation [155]. This calcium/Piezo2-RhoA signaling axis effectively enhances the responsiveness of mesenchymal stem cells (MSCs) to the mechanical microenvironment, promoting their differentiation along osteogenic and chondrogenic lineages. Xu et al. [156] developed a biomimetic ES system capable of reconstructing the resting potential when the host is at rest. During host activity, the system generates biofeedback action potentials in real time, activating the intracellular mechanosensitive protein Piezo1. This process promotes calcium signaling and intracellular mechanotransduction pathways, enhancing the proliferation and migration of BMSCs and human umbilical vein endothelial cells (HUVECs) while simultaneously fostering the osteogenic differentiation of BMSCs and the angiogenesis of HUVECs.

Furthermore, calcium signaling orchestrates cellular transcriptional regulation and energy metabolism. Elevated intracellular calcium concentrations initiate multiple key signaling cascades that directly regulate specific gene expression. Research by Li et al. [105] revealed that endogenous electrical signals transmitted by conductive scaffolds activate VGCCs, triggering Ca^2+^ influx. This influx subsequently activates intracellular signaling cascades, leading to the substantial up-regulation of key osteogenic transcription factors and marker genes, such as type I collagen (COL1A1), Osterix (Osx), and alkaline phosphatase (ALP), thereby driving the osteogenic differentiation of BMSCs. Additionally, bioelectricity generated by electroactive dressings under negative pressure promotes calcium ion influx. Upon binding to calmodulin, these ions activate Ca^2+^/calmodulin-dependent kinase II, which subsequently activates PI3K [157]. This initiates the PI3K/Akt pathway, enhancing osteoblast proliferation, migration, and differentiation. Notably, the regulatory scope of bioelectricity transcends simple differentiation induction to encompass metabolic reprogramming and microenvironment remodeling. Qin et al. [45] discovered that high intracellular Ca^2+^ levels induced by electric fields activate Nrf2 via the Ca^2+^/CaMKKβ/AMPK pathway. This process substantially improves mitochondrial biogenesis and dynamics, specifically the balance between fission and fusion, thereby enhancing the proliferation, migration, and angiogenic potential of HUVECs to accelerate wound healing. Wang et al. [158] demonstrated that electroactive materials activate the Ca^2+^-dependent PI3K/Akt-Nrf2 anti-inflammatory signaling pathway by up-regulating macrophage calcium channel proteins. This activation not only promotes macrophage recruitment and polarization toward the anti-inflammatory M2 phenotype but also recruits Schwann cells and promotes their maturation via paracrine growth factor regulation. Consequently, this establishes a synergistic immune-neural microenvironment conducive to nerve regeneration.

### Regulating cell behavior using endogenous cell adhesion

At the nexus of materials science and cell biology, the initial adhesion of cells to a substrate governs their subsequent spreading, migration, and proliferation while directly influencing cell survival and tissue regeneration. Consequently, the precise regulation of cell adhesion via the chemical and physical characteristics of material interfaces has emerged as a pivotal concern in biomaterials design. Polyphenolic materials have garnered widespread attention recently due to their unique catechol and trihydroxy moieties, exceptional interfacial adhesion, and tunable physicochemical properties [113]. Current literature indicates that these active groups facilitate the adsorption of serum proteins, such as fibronectin (Fn), and the activation of integrin signaling, thereby substantially enhancing cell adhesion and mechanical coupling at the material interface [113,159]. Therefore, polyphenols can serve not only as functional platform materials but also as key molecular modules for regulating cell adhesion behavior, providing effective design strategies for tissue engineering materials and in vivo implants to improve and enhance their ability to regulate cellular behavioral function (Fig. 12B).

The core mechanism by which polyphenols regulate cell adhesion and functional fate originates from the highly biocompatible active interfaces established on material surfaces [160]. Leveraging the chemical versatility of catechol or pyrogallol moieties, polyphenols efficiently adsorb key adhesion proteins, such as Fn, from complex physiological environments via diverse noncovalent interactions [161]. These interactions include hydrogen bonding, hydrophobic effects, π–π stacking, and cation–π interactions. This targeted protein immobilization increases surface ligand density and frequently induces conformational changes that expose previously cryptic cell recognition sites, such as arginine–glycine–aspartic acid (RGD) motifs [162]. Subsequently, integrin receptors on the cell membrane specifically recognize and bind to these exposed RGD sequences [163,164].

This ligand–receptor binding event directly triggers the autophosphorylation of focal adhesion kinase (FAK) at tyrosine 397 [159]. Consequently, this recruits downstream signaling molecules that promote focal adhesion maturation and aggregation while driving the reorganization and spreading of the actin cytoskeleton. Studies utilizing PDA coatings and polyphenol hydrogels demonstrate that polyphenol interfaces substantially promote Fn deposition and conformational activation [165,166]. This process enhances the integrin-mediated FAK phosphorylation signaling cascade rather than directly stimulating the cytoskeleton. This mechanism serves as the molecular basis for driving efficient MSCs migration and stable HUVEC adhesion. Crucially, this stable cell adhesion translates into downstream biological effects by substantially up-regulating the expression of osteogenic markers, such as ALP and osteopontin, alongside angiogenic proteins including vWF and Ang-1 [167]. Collectively, these events enhance cellular proliferation, differentiation, and tissue repair potential.

### Regulating cell behavior using adhesive-electrocoupling synergy

While independent modulation via electrophysical cues or adhesive ligands has demonstrated efficacy, the distinct separation of these modalities limits the potential to fully recapitulate the native tissue microenvironment. Adhesive-electrocoupling materials bridge the gap between “hard” electronic interfaces and “soft” biological systems. This synergy is not merely additive but arises from a reciprocal potentiation mechanism occurring at the cell–material interface. Within the adhesive-electrocoupling hydrogel network, the dynamic electron transfer and redox cycling of polyphenols not only sustain the concentration of catechol groups but also actively participate in the regulation of cellular signaling pathways. The continuous electron exchange equips the hydrogel with potent electron-donating and accepting capabilities, enabling it to actively scavenge excessive ROS and modulate the local oxidative stress microenvironment. This redox-mediated ROS regulation can effectively attenuate pro-inflammatory signaling cascades, thereby promoting macrophage polarization from a pro-inflammatory M1 phenotype to an anti-inflammatory and pro-reparative M2 phenotype. Furthermore, the localized bioelectric coupling facilitated by the conductive network can activate voltage-gated ion channels on cell membranes, directly up-regulating the expression of regeneration-related genes and initiating downstream tissue repair cascades. Therefore, the electron transfer mechanism plays a dual role, providing structural stability through sustained adhesion and actively guiding biological responses through microenvironmental and electrical signal modulation.

Fundamentally, the efficacy of this electro-coupling is predicated on the establishment of a seamless physical interface, where stable adhesion serves as the prerequisite for high-fidelity electrical transduction. Polyphenol-mediated adhesion acts as the physical prerequisite for effective electrical transduction. The polyphenol-mediated adhesion plays an anchoring role. By promoting high-density Fn–integrin binding, the hydrogel minimizes the gap distance at the cell–material junction. This intimate conformational contact minimizes interfacial impedance and suppresses signal attenuation, thereby allowing for the precise delivery of low-voltage electrical stimuli directly to the cell membrane. This proximal contact substantially reduces the effective tunneling barrier and interfacial impedance, thereby facilitating efficient ion–electron transduction and maximizing the charge injection capacity directly into the cellular microenvironment. Without this stable adhesion, charge transfer is often erratic, relying on loose point contacts that necessitate higher voltages, which risks electrochemical damage to the cell.

**Fig. 14.**
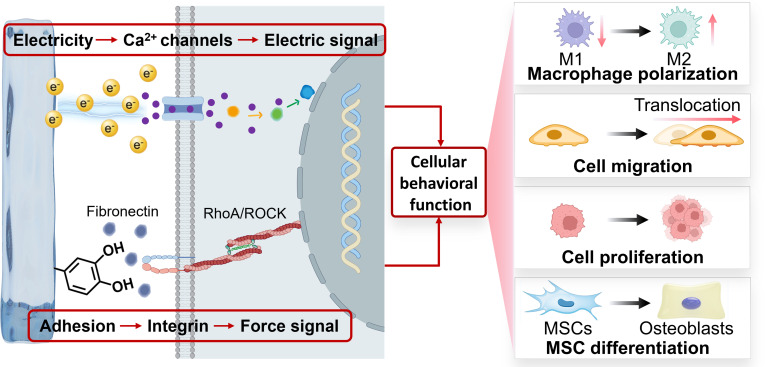
Biological effects of adhesive electrocoupling.

Beyond physical coupling, a sophisticated molecular crosstalk exists between mechanotransduction and electrophysiological signaling. The formation of adhesion plaques triggers the phosphorylation of FAK, which controls cytoskeleton mechanics by regulating the force transmission between the actin cytoskeleton and adhesion plaque proteins, thus putting the cell in a state of “cytoskeleton prestress” [168,169]. This mechanical tension is transmitted to the cell membrane, altering the dynamics of mechanosensitive ion channels such as Piezo1/2 and the kinetics of VGCCs, making them more sensitive to external electric fields [170,171]. Conversely, ES-induced Ca^2+^ influx promotes rearrangement of the actin cytoskeleton and distribution of focal adhesions, further enhancing cell adhesion [172,173]. This synergistic orchestration ultimately reconstructs a high-fidelity bio-interface, driving rapid tissue maturation and functional integration. Facilitated by the mechanical reinforcement derived from stable adhesion, sustained intracellular Ca^2+^ influx drives metabolic reprogramming associated with tissue regeneration and specific gene expression. This process establishes a robust and interactive positive feedback loop linking cellular electrophysiological conduction with cell matrix adhesion. The establishment of this mechanism not only accelerates the tissue repair process but also signifies a paradigm shift in strategies coupling adhesion and electricity. This transition moves from traditional passive regulation to the active and bidirectional dynamic control of cell fate. In summary, polyphenol-mediated adhesive-electrocoupling effectively modulates cellular behavior through synergistic mechanisms (Fig. 14).

## Adhesive-Electrocoupling Hydrogels for Tissue Repair

### Bone tissue repair

Bone tissue injury is one of the pressing challenges in clinical practice, commonly seen across all age groups [174]. For large bone defects, relying solely on the cellular function of the defect site is insufficient. Hydrogels with highly hydrophilic 3D network structures have been widely reported for the treatment of skeletal disorders, serving as scaffolds for bone tissue engineering, drug delivery carriers, and materials for bone regeneration and repair [175]. Among them, adhesive-electrocoupling hydrogels demonstrate excellent potential for bone tissue regeneration due to their ability to form well-integrated interfaces with defect sites, regulate the immune microenvironment and cell behavior, and couple with endogenous electric fields within bone tissue.

To further enhance osteogenic efficiency, strategies that introduce active electrical microenvironments via stimuli-responsive materials have emerged as a potent therapeutic approach. Zhou et al. [176] developed an injectable nanocomposite hydrogel featuring superior conformability, robust bone adhesion, and ultrasound-responsive properties to electrically accelerate bone healing. The material’s exceptional adhesive strength facilitates tight interfacial bonding with bone, enabling it to effectively bridge separated porcine bone fragments. Upon ultrasound, the hydrogel generates a controllable electrical output that augments the osteogenic differentiation of BMSCs. This mechanism functions by increasing intracellular calcium influx and up-regulating the PI3K/Akt and MAPK kinase (MEK)/ERK signaling pathways, thereby substantially enhancing osteogenesis both in vitro and in vivo and offering a novel therapeutic strategy for irregular bone defects. Conversely, Wang et al. [177] developed a flexible ES system for bone regeneration comprising a novel flexible hybrid tribo/piezoelectric nanogenerator (HTP-NG) and a conductive injectable hydrogel. This integrated system establishes an osteogenic microenvironment at the defect site by modulating multiple biological processes. The HTP-NG is implanted subcutaneously in the thigh, where it harvests mechanical energy from knee joint movement to generate biphasic electrical pulses; these pulses are subsequently transmitted to the conductive hydrogel for stimulation therapy. Notably, the hydrogel exhibits robust adhesion to diverse substrates, which effectively minimizes interfacial impedance with the surrounding tissue. Under ES, the conductive hydrogel substantially enhances cell proliferation, migration, and adhesion, ultimately promoting osteogenic differentiation both in vitro and in vivo.

**Fig. 15.**
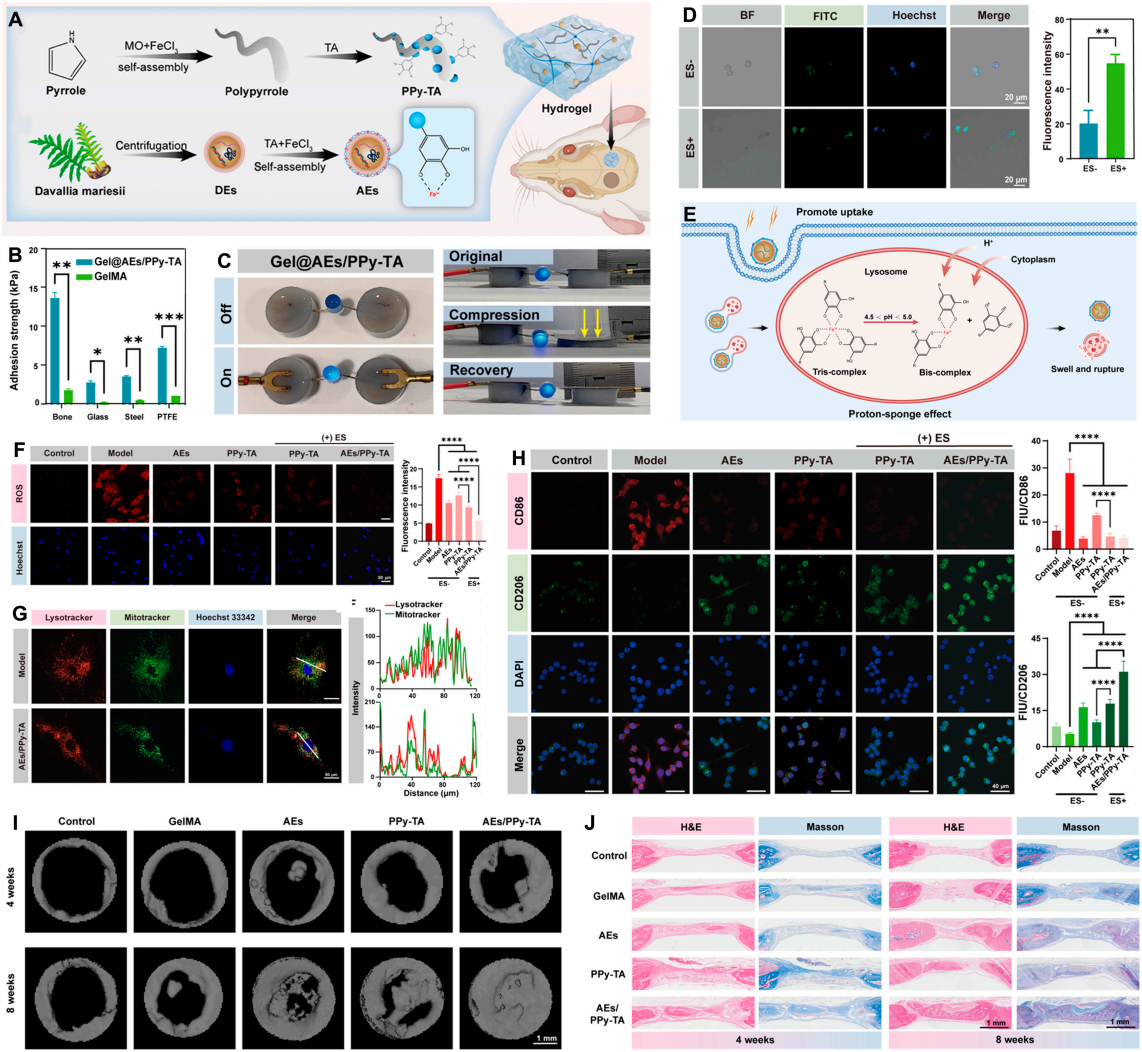
Adhesive-electrocoupling hydrogels for bone tissue repair. Reproduced with permission from [178]. Copyright 2025, American Chemical Society. (A) Preparation strategy of GelMA@AEs/PPy-TA adhesive-electrocoupling hydrogel. (B) Adhesion strength of hydrogel to different materials. (C) Hydrogel integration into the circuit and maintaining LED illumination during compression and release cycles. (D) Fluorescence images of AEs in RAW 264.7 display the signals of nuclei (blue) and AEs (green) and immunofluorescence staining quantification analysis. (E) Schematic illustration of the mechanism of improving the efficiency of AE absorption. (F) Fluorescence images and quantitative analysis of intracellular ROS-scavenging performance of RAW 264.7 on the various hydrogels. (G) Fluorescence images of hydrogel in BMSCs displayed the signals of lysosomes (red), nuclei (blue), and mitochondria (green). (H) Immunofluorescence images and quantitative analysis of macrophages CD86 (M1) and CD206 (M2) on different hydrogels. (I) Computed tomography (CT) images of calvaria samples from 4- and 8-week post-surgery groups. (J) Hematoxylin and eosin (H&E) and Masson’s trichrome staining of samples at 4 and 8 weeks.

Furthermore, the synergistic integration of adhesive-electrocoupling hydrogels with bioactive cargo delivery systems offers a sophisticated platform for modulating the osteogenic microenvironment. Zheng et al. [178] incorporated a delivery system composed of metal-polyphenol network (MPN)-armored exosomes (AEs) and a TA-mediated polypyrrole (PPy-TA) conductive fiber network into a GelMA hydrogel matrix to develop a GelMA@AEs/PPy-TA adhesive-electrocoupling hydrogel for bone regeneration (Fig. 15A). The phenolic hydroxyl groups in TA endowed the hydrogel with strong adhesion to various substrates, particularly bone tissue, achieving a maximum adhesion strength of 13.6 kPa (Fig. 15B). The conductive properties of PPy enabled efficient charge transport, allowing the hydrogel to be integrated into the circuit while keeping the light-emitting diode (LED) lit, which remained illuminated during compression and release cycles, highlighting the hydrogel’s excellent dynamic conductivity and mechano-electrical stability (Fig. 15C). Meanwhile, the MPN armor on the AEs protected them from ROS-induced degradation in inflammatory microenvironments. The strong tissue adhesion allowed the hydrogel to efficiently harness endogenous bioelectricity, enhancing cellular uptake of exosomes (Exos) (Fig. 15D). Beyond protection from ROS, the MPN shell also facilitated lysosomal escape of Exos via the proton sponge effect (Fig. 15E), thereby preserving their therapeutic payloads and maximizing their osteoinductive potential.

The good electrical conductivity promoted electron transfer to TA, maintaining the redox state between catechol and quinone groups, ensuring a continuous supply of antioxidant phenolic hydroxyls. This dynamic redox activity conferred long-term antioxidant capability, which was crucial for scavenging ROS (Fig. 15F), restoring mitochondrial function (Fig. 15G), and polarizing macrophages toward the anti-inflammatory M2 phenotype (Fig. 15H), thus creating a regenerative microenvironment. This multifunctional hydrogel integrates bioelectric modulation, sustained antioxidation, and enhanced exosome delivery to provide a comprehensive platform for bone regeneration, effectively promoting new bone formation in a rat calvarial defect model (Fig. 15I and J).

Hydrogels have been extensively investigated for their potential in cartilage repair [179]. Rheumatoid arthritis (RA) is a prevalent chronic autoimmune disease characterized by compromised lubrication, inflammatory infiltration, and cartilage degeneration. Consequently, long-term improvements in joint lubrication, the reduction of inflammation, and the repair of damaged cartilage are critical for effective RA treatment. He et al. [180] proposed an injectable bioadhesive lubricating hydrogel comprising a DA-modified hyaluronic acid (DA-HA) network, a sulfonated hyaluronic acid (SO_3_^−^-HA) network, and a kartogenin (KGN)-grafted DA-hybridized graphene quantum dot-supported Cu single-atom nanozyme (DAGQD@Cu@KGN SAN). The incorporation of DA imparts bioadhesive properties, enabling the hydrogel to persist within the joint cavity for extended periods. Meanwhile, SO_3_^−^ inherently reduces friction by featuring a sulfonate group, naturally binding water to hydrate surfaces. The SO_3_^−^-HA network enhances lubrication upon contact with the cartilage surface, effectively preventing wear by reducing the coefficient of friction in RA models. By adjusting the ratio of SO_3_^−^-HA to DA-HA, the hydrogel achieves a synergistic balance between lubrication and bioadhesion. Furthermore, the DAGQD@Cu@KGN SAN component exhibits highly efficient enzyme-like ROS-scavenging activity, which alleviates the inflammatory microenvironment within the joint cavity. Mimicking the complex adhesion mechanism of mussels, the hydrogel facilitates electron transfer from the Cu SAN to quinone groups in the DAGQD@Cu SAN, maintaining the redox balance between quinone and catechol groups within the network, and achieving adhesive-electrocoupling. This mechanism preserves sufficient catechol groups to ensure durable adhesion. Ultimately, this injectable bioadhesive lubricating hydrogel not only prevents cartilage wear and provides sustained antioxidant and anti-inflammatory effects in early-stage RA but also repairs damaged cartilage in late-stage RA, offering a promising full-cycle strategy for RA treatment.

### Periodontal tissue repair

Periodontitis leads to the destruction of tooth-supporting tissues, presenting a complex challenge due to the hierarchical soft–hard tissue interface within a humid, bacteria-rich oral environment [181]. Conventional therapies often fail to achieve stable fixation or coordinated multi-tissue regeneration. Adhesive-electrocoupling hydrogels offer a promising solution by providing robust wet adhesion to the tooth root and bone surfaces. Furthermore, by mimicking the electrophysiological microenvironment, these materials couple with endogenous bioelectric signals to modulate immune responses and promote periodontal ligament stem cell differentiation, ultimately facilitating the reconstruction of the physiological tooth–bone attachment.

Enhancing the transduction of electrical signals at the cell–matrix interface through robust adhesion and conductivity is critical for activating intracellular regenerative pathways. Li et al. [105] prepared a composite PGO-PHA-AG adhesive-electrocoupling hydrogel scaffold by incorporating PGO and hydroxyapatite nanoparticles (PHA) into an alginate and gelatin (AG) network featuring dual physiochemical crosslinking. This scaffold exhibited synergistic capabilities, including conductivity, ROS scavenging, anti-inflammatory properties, and immunomodulation. By utilizing the cellular affinity of PDA, the scaffold promoted the adhesion and spreading of BMSCs. This effect potentially arises from the activation of cytoskeletal tension via the RhoA and ROCK signaling pathway, which subsequently enhances osteogenic differentiation. Enhanced cell adhesion further facilitated the transmission of endogenous electrical signals to cells, thereby activating calcium channels and promoting Ca^2+^ influx. The combined electrical and chemical cues provided by the scaffold up-regulated the expression of osteogenic genes, specifically COL1A1, Osx, and ALP in BMSCs, driving their differentiation toward the osteogenic lineage. Alizarin Red S staining revealed a greater number of mineralized nodules in the scaffold group subjected to ES. In a model of periodontal bone defects in diabetic rats, this platform induced robust bone regeneration.

**Fig. 16.**
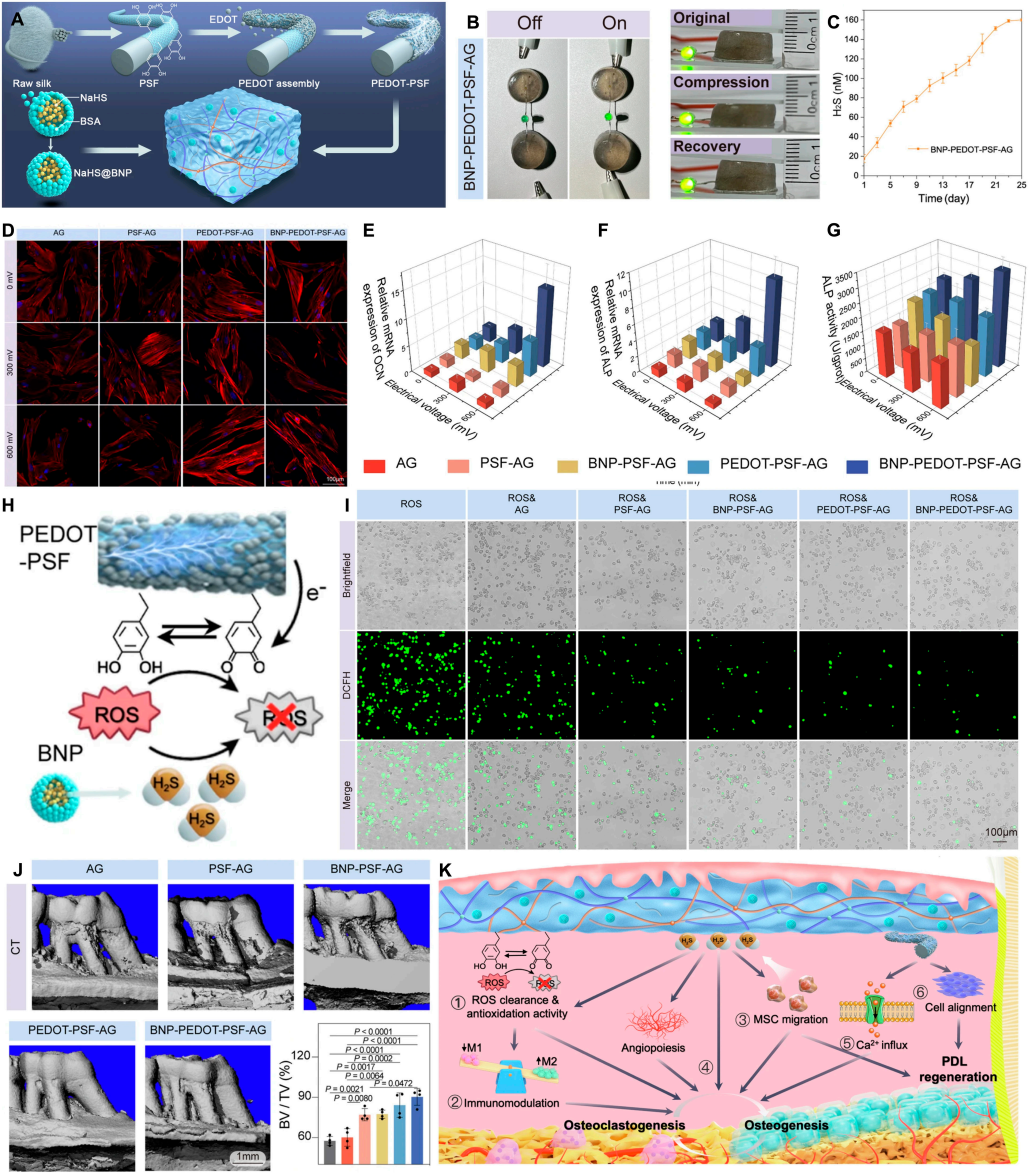
Adhesive-electrocoupling hydrogels for periodontal tissue repair. Reproduced with permission from [182]. Copyright 2024, Springer Nature. (A) Preparation strategy of BNP-PEDOT-PSF-AG adhesive-electrocoupling hydrogel. (B) Hydrogel scaffold integration into the circuit and maintaining LED illumination during compression and release cycles. (C) Amount of H_2_S released from the BNP-PEDOT-PSF hydrogel in 25 d. (D) Cell morphologies of PDLSCs on the surfaces of different hydrogel. (E) OCN expression of PDLSCs on different hydrogels under various ES potentials. (F) ALP activity of PDLSCs on different hydrogels under various ES potentials. (G) ALP expression of PDLSCs on different hydrogels under various ES potentials. (H) Antioxidative mechanism of the BNP-PEDOT-PSF-AG hydrogel. (I) Intracellular ROS-scavenging performance of THP-1 on the various hydrogels. (J) Micro-CT images of the rat maxillary first molar at the site of implantation and quantification analysis of bone volume to tissue volume ratio (BV/TV). (K) Schematic illustration of the mechanism of the BNP-PEDOT-PSF-AG hydrogel in promoting integrated periodontium regeneration under diabetic periodontic conditions.

Beyond direct ES, integrating the sustained release of bioactive gas transmitters with conductive networks offers a synergistic approach to modulate the regenerative microenvironment. Fang et al. [182] developed a polyphenol-mediated redox-active BNP-PEDOT-PSF-AG adhesive-electrocoupling hydrogel by combining conductive PEDOT-assembled PDA-mediated silk microfiber (PEDOT-PSF) with a sustained-release system constructed from NaHS-encapsulated bovine serum albumin nanoparticles (BNP) (Fig. 16A). PEDOT-PSF confers excellent conductivity upon the hydrogel, allowing it to integrate into the circuit and keep the LED illuminated. Moreover, the LED maintained its luminescence throughout repeated compression and release cycles, demonstrating the superior dynamic conductivity of the hydrogel (Fig. 16B). Furthermore, this hydrogel demonstrated the capacity to release H_2_S continuously for up to 21 d (Fig. 16C). BNP-PEDOT-PSF-AG hydrogel promoted the spreading and adhesion of human-derived periodontal ligament stem cells (PDLSCs) (Fig. 16D). Upon application of ES to simulate endogenous electric fields, the aspect ratio of PDLSCs on the hydrogel increased from 3.04 ± 0.16 to 4.93 ± 0.45 at 600 mV. Under ES, the PEDOT-PSF-AG group exhibited substantially up-regulated OCN expression (Fig. 16E) and ALP levels (Fig. 16F), concurrent with enhanced ALP activity (Fig. 16G), thereby substantiating the osteoinductivity of PEDOT-PSF. Electrons transfer from PEDOT to PDA within the PEDOT-PSF complex. This process works in synergy with H_2_S to promote dynamic redox reactions between catechol and quinone groups, thereby conferring redox activity to the hydrogel (Fig. 16H). BNP-PEDOT-PSF-AG hydrogel can effectively reduce the intracellular ROS level of THP-1 cells (Fig. 16I).

At 4 weeks post-treatment in diabetic periodontitis rats, the BNP-PEDOT-PSF-AG group displayed the highest bone volume fraction, indicative of substantial periodontal tissue repair (Fig. 16J). The superior regenerative efficacy of this hydrogel in treating diabetic periodontal defects is attributed to a multifaceted mechanism orchestrated by the synergistic action of PEDOT-PSF and H_2_S (Fig. 16K). Biochemically, the system efficiently scavenges ROS to alleviate local inflammation and oxidative stress, thereby attenuating osteoclastogenesis while regulating macrophage polarization through lipid metabolism modulation. At the cellular level, the sustained release of H_2_S facilitates the timely recruitment of MSCs, which, aided by PSF-mediated adhesion, ensure robust angiogenesis and activate bone formation via the promotion of autophagy in PDLSCs. Furthermore, the superior conductivity of the hydrogel enables the transmission of endogenous bioelectricity to trigger calcium ion influx for in situ osteogenesis and improves cellular alignment, ultimately culminating in functional periodontal ligament regeneration.

Bacteria-driven periodontal inflammation is intimately associated with early cognitive impairment. Periodontal pathogens can induce neuropathology via hematogenous and neural pathways along the oral brain axis [183]. Epidemiological data indicate that patients with periodontitis face a 77% higher risk of developing cognitive impairment compared to those without the condition, creating a vicious cycle that impedes tissue repair [184]. Consequently, restoring oral microbial homeostasis represents a pivotal strategy for delaying or preventing neurodegenerative diseases. Yang et al. [185] engineered the phenolic hydroxyl groups of dihydrocaffeic acid (DHCA)-anchored zinc ions onto the surface of Fe-porphyrin metal–organic framework (Fe-TCPP) to form bimetallic Fe-TCPP-DHCA-Zn via chelation. This complex was subsequently integrated into a hydrogel matrix composed of oxidized dextran, DHCA-grafted chitosan, and gelatin to fabricate the Fe-TCPP-DHCA-Zn/ODG hydrogel. This DHCA-mediated bimetallic strategy augments the sonodynamic properties of Fe-TCPP by narrowing the band gap, effectively boosting antibacterial activity. Simultaneously, the Fe^2+^/Fe^3+^ redox couple in Fe-TCPP-DHCA-Zn interacts with polyphenols to facilitate phenolic quinone conversion via multi-electron transfer, thereby preserving a high concentration of catechol moieties within the hydrogel for extended periods. The hydrogel establishes a microenvironment conducive to periodontal tissue regeneration by supporting anti-inflammatory and immunomodulatory processes. DHCA scavenges ROS to alleviate oxidative stress and regulate local immune responses, while the released Zn^2+^ ions inhibit osteoclastic bone resorption and promote osteoblastic bone formation. Leveraging its enhanced sonodynamic efficacy, the hydrogel effectively eradicates subgingival pathogens. Moreover, DHCA and its degradation byproducts protect and modulate beneficial bacteria, thereby maintaining oral flora homeostasis. In a diabetic periodontitis rat model, the Fe-TCPP-DHCA-Zn/ODG hydrogel not only enhances periodontal tissue regeneration but also ameliorates behavioral and cognitive deficits. This is achieved by preserving oral microbial homeostasis, down-regulating systemic inflammation, and mitigating neuroinflammation. Single-cell analysis identified macrophage fibroblast interactions as a driving factor in periodontal regeneration under diabetic conditions. Ultimately, this hydrogel system demonstrates a dual therapeutic effect, promoting both periodontal soft and hard tissue remodeling while alleviating anxiety-like behaviors via the oral–brain axis, thereby offering novel insights into microbiome–brain interactions.

### Skin tissue repair

The skin, the largest organ in the human body, performs a wide range of physiological functions, including sensation, absorption, excretion, metabolism, and immune defense. As the first line of defense between the body and the external environment, it is highly vulnerable to factors such as mechanical damage, infection, and irritation [36]. When the skin barrier is compromised, the body initiates a rapid and complex repair process involving inflammatory responses, cellular metabolic regulation, cell migration, tissue proliferation, and remodeling to restore tissue homeostasis and repair the barrier. Adhesive-electrocoupling hydrogels can closely adhere to wounds and interact with the wound’s endogenous electric field, thereby promoting skin wound healing by modulating the immune environment and cellular functions.

Establishing a seamless bio-interface is prerequisite for effective signal transduction and therapeutic intervention, prompting the development of hydrogels with optimized adhesive and conductive properties. Wei et al. [186] engineered a multifunctional hydrogel featuring conductive, adhesive, and photothermal properties. By establishing a robust and seamless interface with skin tissue, the material minimizes interfacial impedance, thereby enabling real-time, high-fidelity acquisition of physiological signals. Concurrently, this superior interfacial contact enhances photothermal transduction efficiency; the resulting localized hyperthermia effectively inhibits bacterial proliferation, ultimately facilitating tissue regeneration. Similarly addressing complex healing environments, Yang et al. [187] developed a multifunctional hydrogel exhibiting conductive, adhesive, and antibacterial properties to mitigate the chronic inflammation and insufficient vascularization associated with diabetic wounds. The material’s superior tissue adhesiveness ensures robust interfacial integration with the wound site, while its broad-spectrum antibacterial activity effectively suppresses infection-driven inflammation. Furthermore, the hydrogel’s conductivity facilitates the restoration of endogenous electric fields and regulates the local microenvironment. These features collectively promote cell migration, angiogenesis, and collagen deposition, thereby accelerating tissue repair. In a diabetic rat model, the treatment demonstrated remarkable therapeutic efficacy, achieving a wound closure rate of 98% by day 15.

**Fig. 17.**
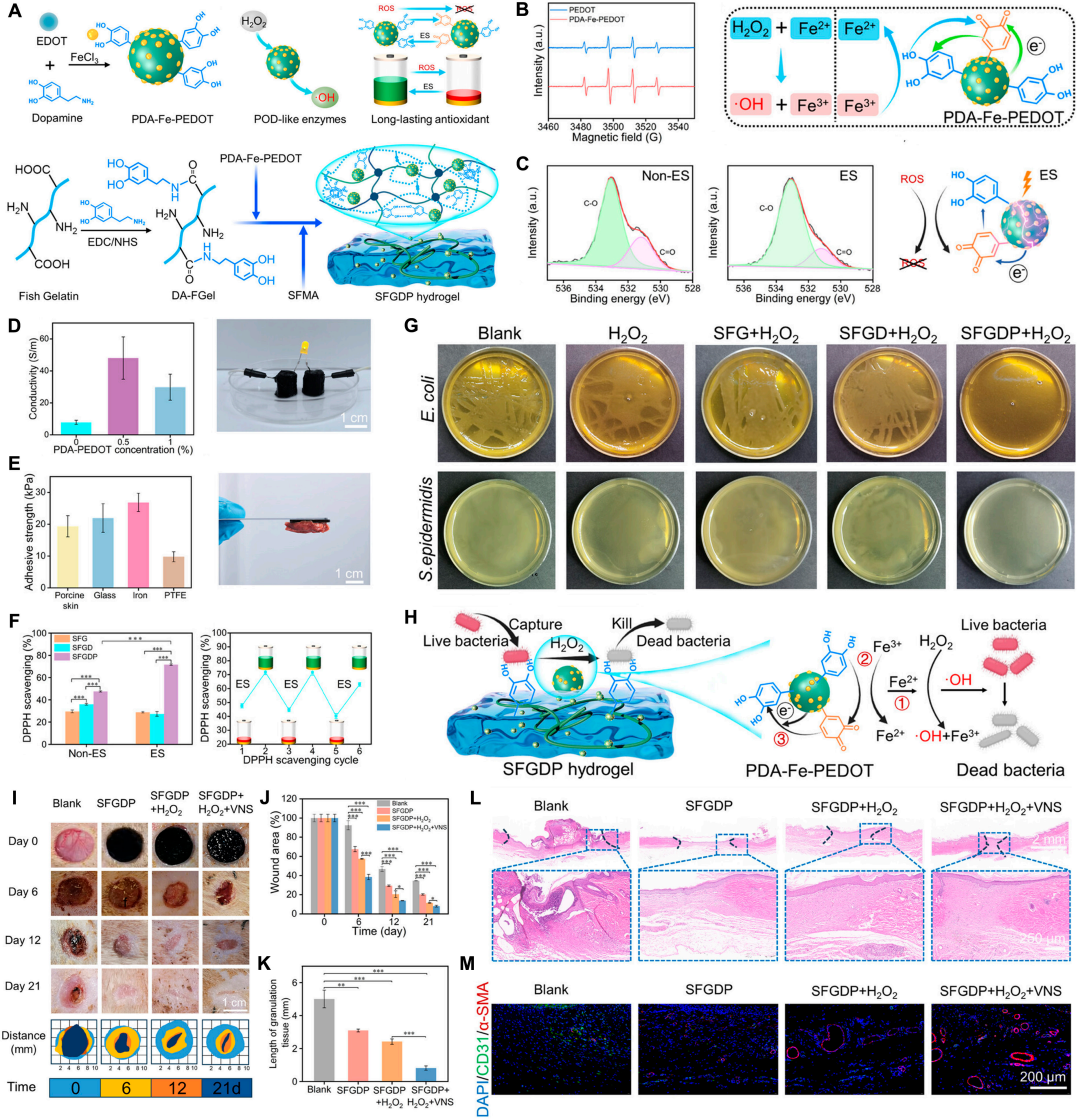
Adhesive-electrocoupling hydrogels for skin tissue repair. Reproduced with permission from [188]. Copyright 2025, Wiley-VCH. (A) Preparation strategy of SFGDP adhesive-electrocoupling hydrogel. (B) ESR diagram of PDA-Fe-PEDOT conductive nanozymes and their strong POD enzyme activity mechanism. (C) X-ray photoelectron spectroscopy analysis of PDA-Fe-PEDOT conductive nanozymes before and after ES, and its long-term antioxidant mechanism. (D) The conductivity of the hydrogel and its connection to the circuit maintain the LED illumination. (E) Adhesion strength of SFGDP hydrogel to different substrates and its adhesion to wet tissues. (F) Comparison of DPPH scavenging capacity of different hydrogels with and without ES, and restoration of antioxidant capacity of SFGDP hydrogels by ES. (G) Antibacterial effect of SFGDP hydrogel against *E. coli* and *S. epidermidis*. (H) Diagram illustrating the antibacterial mechanism. (I) Representative digital images of the wound healing process in different treatment groups. (J) Quantification of the wound closure area at different time intervals. (K) Quantify granulation tissue length. (L) Representative H&E staining images of wound samples in the different groups on day 21. (M) Fluorescence staining of vascular markers CD31 and α-SMA (α-smooth muscle actin) in wound tissue after 21 d of treatment across different groups.

To further enhance the antioxidative capacity and electroactivity of hydrogels, redox-active nanomaterials have been introduced to construct catalytic interfaces for accelerated healing. Ran et al. [188] developed a polyphenol-conductive nanozyme-enhanced SFGDP adhesive-electrocoupling hydrogel, which includes methacrylated silk fibroin (SFMA), DA-grafted fish gelatin (FGel-DA), and DA-mediated PEDOT (PDA-Fe-PEDOT) conductive nanozyme (Fig. 17A). This hydrogel, when combined with vagus nerve ES, offers a comprehensive solution for diabetic wound repair. The polyphenol-conductive nanozyme, synthesized via DA-mediated EDOT polymerization under FeCl_3_-induced oxidation conditions, exhibits high peroxidase-like (POD) activity and catalyzes the degradation of H_2_O_2_ into ·OH. In electron spin resonance (ESR) experiments using 5,5-dimethyl-1-pyrroline N-oxide as a trap, the PDA-Fe-PEDOT conductive nanozyme produces a substantially stronger free radical signal. This effect is attributed to the DA in the PDA-Fe-PEDOT structure, which enhances its electron-donating ability, promoting the reduction of Fe^3+^ to Fe^2+^ and maintaining the POD catalytic cycle (Fig. 17B). The PDA-Fe-PEDOT nanozyme also responds to ES. After electrochemical treatment, the content of C=O groups decreases, while the C–O groups increase, and quinone groups are reduced to catechol groups (Fig. 17C). The PDA-Fe-PEDOT conductive nanozyme enhances the hydrogel’s conductivity, allowing it to integrate into the circuit and keep the LED illuminated (Fig. 17D). Additionally, the abundant catechol groups in the hydrogel substantially improve its adhesion to pigskin, enabling it to firmly adhere to moist soft tissue (Fig. 17E).

Because of the electron transfer effect in adhesive-electrocoupling hydrogels, these hydrogels demonstrate recoverable 2,2-diphenyl-1-picrylhydrazyl (DPPH) radical scavenging ability by reducing quinone groups back to phenolic hydroxyl groups (Fig. 17F). Furthermore, the developed polyphenol-conductive nanozyme-enhanced adhesive-electrocoupling hydrogels show strong antibacterial activity against both Gram-positive *Staphylococcus epidermidis* and Gram-negative *Escherichia coli* bacteria (Fig. 17G). This antibacterial effect is attributed to the polyphenolic components in the hydrogel, which can reduce Fe^3+^ to Fe^2+^, ensuring the continuous generation of free radicals. Additionally, the reduction of quinone groups to phenolic groups via electron transfer maintains the redox balance of DA, contributing to highly efficient antibacterial activity (Fig. 17H). In a diabetic rat wound model infected with *E. coli*, the polyphenol-conductive nanozyme-enhanced adhesive-electrocoupling hydrogels, combined with ES, demonstrated excellent wound healing effects (Fig. 17I). These effects included a substantial reduction in wound area (Fig. 17J), granulation tissue width (Fig. 17K), and a skin structure similar to normal tissue (Fig. 17L), as well as substantial angiogenesis (Fig. 17M).

Inflammation and depression represent severe complications of diabetes [189]. Their interaction establishes a feedback loop that potentially impedes diabetic wound healing. Hou et al. [12] proposed a combined therapeutic approach utilizing conductive hydrogel microneedles loaded with DA and *Lycium barbarum* polysaccharide in conjunction with electroacupuncture stimulation at the Dazhui acupoint (GV14). Stimulation of GV14 via electroacupuncture inhibits systemic inflammation through the vagus adrenal axis while elevating 5-hydroxytryptamine levels. This process effectively alleviates depressive symptoms in rats with diabetic wounds. The conductive hydrogel microneedles respond to external ES from electroacupuncture by converting dopaquinone into DA catechol via electron transfer. This conversion promotes local immune regulation, continuous free radical scavenging, and macrophage phenotype modulation. By up-regulating anti-inflammatory factors and down-regulating pro-inflammatory cytokines, the system exhibits potent and sustained anti-inflammatory and antioxidant effects. Furthermore, the polyphenol conductive hydrogel microneedles responded to ES by promoting the adhesion and spreading of RSC96 Schwann cells, thereby facilitating neurological function recovery. Consequently, this accelerated the healing of both wound tissue and peripheral nerves. While modulating local and systemic inflammation, this strategy simultaneously alleviated depressive-like behaviors in diabetic rats. Thus, it offers a novel clinical perspective for treating diabetes-related comorbidities and mood disorders.

In another study, Jia et al. [106] designed a mussel-inspired nanozyme by in situ reducing ultrasmall Ag NPs with TA-chelated Ag. The TA–Ag nanozyme retains abundant phenolic hydroxyl groups, maintaining the dynamic redox balance of phenolic quinones. Its incorporation into hydrogels imparts adhesive-electrocoupling properties, similar to the long-lasting, reusable adhesion of mussels. The phenolic TA–Ag nanozyme is uniformly distributed within the hydrogel and interacts with the hydrogel network, enhancing both its mechanical properties and conductivity. This makes it suitable as an adhesive bioelectrode for detecting physiological signals. Furthermore, the hydrogel binds to surrounding tissues, acting as a wound repair patch and accelerating wound regeneration in rats. Additionally, a PDA-assisted extraction and protection process was employed to prepare ultralong microfilaments (PDA-mSF) [190]. Subsequently, PEDOT was assembled onto the outer surface of the microfilaments using catechol groups as adhesion sites, resulting in ultralong PEDOT-coated PDA-functionalized microfilaments with a core–sheath structure (PEDOT-PDA-mSF). An electrocoupling patch was then constructed based on the PEDOT-PDA-mSF microfilaments. The PDA in the patch possesses electron-trapping capabilities that scavenge ROS, converting catechols into quinones. The conductive PEDOT-PDA-mSF facilitates electron injection from PEDOT into PDA, reducing quinones back to catechols. Thus, the dynamic redox behavior between quinones and catechols imparts high ROS-scavenging performance to the PEDOT-PDA-mSF. The PDA in the patch also provides cell/tissue affinity and adhesion, promoting better cell attachment and migration. The patch’s excellent conductivity creates a conductive pathway for endogenous bioelectrical transport to the wound, guiding cell migration, collagen deposition, and alignment. Additionally, the patch alleviates oxidative stress through its long-lasting ROS-scavenging properties, promoting fibroblast activity, regulating collagen deposition, and modulating extracellular matrix remodeling by down-regulating matrix metalloproteinase-2 and matrix metalloproteinase-9 expression. These combined effects synergistically accelerate diabetic wound healing.

### Myocardial tissue repair

Following MI, the infarcted myocardium is gradually replaced by poorly conductive scar tissue, leading to arrhythmias and substantial cardiac remodeling [56]. Therefore, there is an urgent need to explore novel therapeutic strategies to reduce CM loss and restore the complex architecture of myocardial tissue after infarction. Adhesive-electrocoupling hydrogels can firmly adhere to damaged myocardial tissue and reconstruct electrical signal conduction, enabling electrocoupling therapy to mitigate arrhythmias and limit ventricular remodeling caused by prolonged heterogeneous conduction.

Developing hydrogels that can accommodate the cyclic deformation of the myocardium while maintaining stable electrical conduction remains a formidable challenge in cardiac tissue engineering. Yu et al. [191] developed an injectable mechanical-electrical coupling hydrogel utilizing dynamic covalent and noncovalent crosslinking. This hydrogel is designed for minimally invasive delivery into the pericardial cavity. Exhibiting superior wet adhesion, the hydrogel establishes a stable interface with tissue, enabling highly compliant coupling with the dynamically beating myocardium. Furthermore, its conductivity matches that of native myocardial tissue, thereby bridging the electrical gap between healthy and infarcted regions to enhance conduction velocity and synchronization. When combined with cell therapy, this system effectively attenuates ventricular fibrosis and remodeling, promotes angiogenesis, restores electrical propagation, and facilitates the clinical translation of cardiac tissue engineering. In addition, Lee et al. [192] developed a multifunctional hydrogel cardiac patch designed to provide mechanical support, electrical conductivity, and tissue adhesiveness for myocardial recovery. The hydrogel exhibits robust adhesion to the myocardium, ensuring stable integration with dynamic cardiac tissues. By matching the elastic modulus of native heart tissue, the hydrogel offers critical mechanical reinforcement that substantially suppresses ventricular remodeling and reduces fibrotic area. Furthermore, the high conductivity of the hydrogel facilitates the re-establishment of electrical propagation, leading to effective myocardial repair. In vivo animal experiments demonstrated that the patch substantially improved cardiac function and mitigated pathological remodeling of the infarcted heart.

**Fig. 18.**
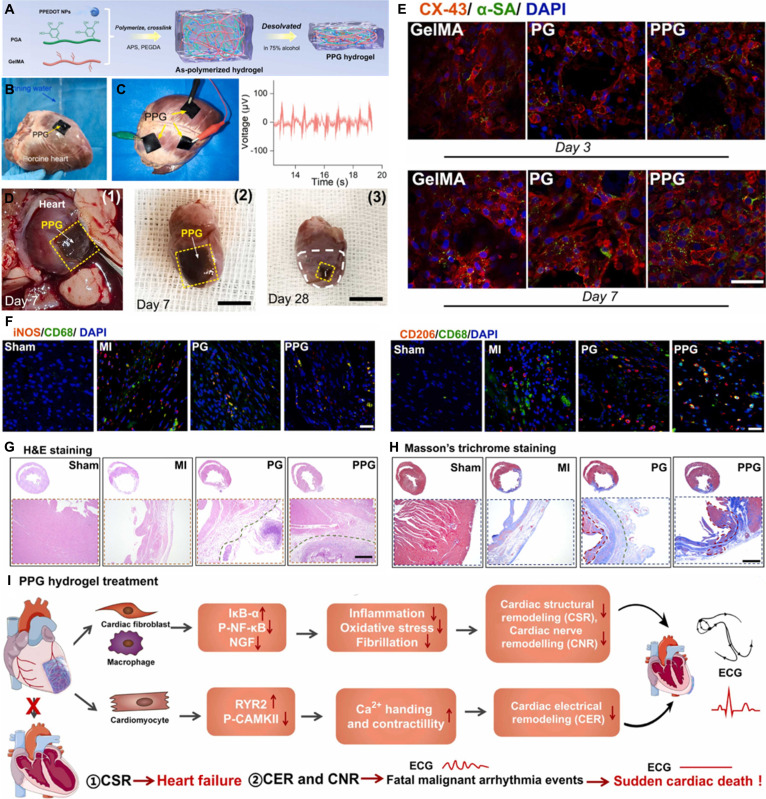
Adhesive-electrocoupling hydrogels for heart tissue repair. Reproduced with permission from [81]. Copyright 2024, Elsevier. (A) Synthesis diagram of PPG adhesive-electrocoupling hydrogel preparation process. (B) PPG adhesive-electrocoupling hydrogel firmly adhered on the porcine heart under water flushing. (C) PPG adhesive-electrocoupling hydrogel as self-adhesive electrodes adhering on a porcine heart to detect applied electromyography (EMG) signals. (D) In vivo wet adhesion ability of PPG adhesive-electrocoupling hydrogel. (E) Immunofluorescent staining of cardiac-specific proteins of CX-43 (red) and α-actinin (green) in the CMs on various hydrogels. (F) Anti-inflammatory and immunomodulatory ability of the PPG adhesive-electrocoupling hydrogel in the infarcted myocardium. (G) Representative images of H&E staining of heart sections in different group at day 28 post-MI. (H) Representative Masson’s trichrome-stained myocardial sections in each group at day 28 post-MI. (I) Potential mechanism of PPG adhesive-electrocoupling hydrogel suppressed ventricular remodeling post-MI.

Beyond providing structural support, the establishment of a robust biointerface between conductive hydrogels and myocardial tissue is paramount for restoring electrical propagation and facilitating the therapeutic repair of infarcted myocardium. Chen et al. [81] developed an adhesive-electrocoupling PPG hydrogel for MI treatment, composed of poly gallic acid (PGA), GelMA, and PDA-hybridized PEDOT NPs (PPEDOT NPs) (Fig. 18A). Utilizing an ethanol desolvation strategy, the hydrogel rapidly absorbs the hydration layer from the cardiac tissue surface, enhancing the interaction between its abundant catechol groups and the tissue to create strong wet adhesion. It adheres tightly to fresh, smooth porcine heart tissue, even after rinsing with running water (Fig. 18B). This strong adhesion allows PPEDOT NPs to reconstruct conduction pathways in the damaged myocardium, forming a stable, seamless, and electrically coupled interface between the visco-electro-coupled PGG hydrogel and the heart. When applied to the moist, curved surface of an isolated porcine heart, the hydrogel can serve as an adhesive electrode to record ECG signals by simulating heartbeat through manual compression (Fig. 18C).

Moreover, the dynamic redox balance established between quinone groups in the PPEDOT NPs and PGA confers long-lasting adhesion, maintaining firm attachment to cardiac tissue even 28 d post-implantation (Fig. 18D). The highly conductive PGG hydrogel enhances the expression of CX-43 in CMs, thereby improving electromechanical coupling (Fig. 18E). Additionally, PGA imparts immunomodulatory properties to the hydrogel by promoting M2 macrophage polarization, resulting in excellent anti-inflammatory effects (Fig. 18F). Implantation of the PGG hydrogel into infarcted hearts substantially reduced MI area and left ventricular fibrosis, ultimately improving cardiac function in MI rat models (Fig. 18G and H). Furthermore, the PGG hydrogel up-regulated the expression of the NF-κB inhibitor α gene. This inhibitor protein retains the key transcription factor NF-κB, which regulates immune response, cell growth, and tissue repair, in its inactive state within the cell, thereby mitigating the harmful effects of prolonged NF-κB activation on cardiac tissue. Simultaneously, the hydrogel down-regulated the expression of nerve growth factor produced by overactivated macrophages and fibroblasts in the infarct zone, reducing cardiac neural remodeling. In addition, the hydrogel suppressed activation of the CaMKII–RyR2 signaling pathway by up-regulating expression of the aspartate β-hydroxylase gene, improving Ca^2+^ signal transduction, and restoring electrical conduction function (Fig. 18I).

### Brain tissue repair

Traumatic brain injury (TBI), caused by mechanical damage, neuroinflammation, and neurodegeneration, has an extremely high mortality rate and can result in disability or death. Survivors of severe TBI often experience shortened life expectancy and permanent functional impairments, collectively imposing a substantial socioeconomic burden. Traditional biopolymer-based hydrogels cannot fully replicate the diverse physiological microenvironmental cues such as mechanical, chemical, structural, and biological signals that govern cell behavior and tissue responses in the brain. Given the crucial role of endogenous electric fields in neural tissue regeneration, hydrogels with conductivity matched to that of brain tissue can effectively mimic interneuronal neurotransmission and help restore the function of damaged neural systems [65]. However, such hydrogels still suffer from mechanical mismatch and poor interfacial integration with soft brain tissue.

In the context of Parkinson’s disease (PD) therapy, Chen et al. [193] developed a fully biodegradable conductive hydrogel engineered to match the shear modulus and conductivity of native brain tissue. Beyond its intrinsic ability to promote NSC proliferation and differentiation in vitro, the hydrogel exhibits potent antioxidant and anti-inflammatory properties. When synergized with acupuncture in a PD rat model, the system elevated serum TGF-β1 and SDF-1 levels, modulated neuroinflammation via the M1-to-M2 microglial transition, and facilitated dopaminergic neuron regeneration, ultimately restoring motor function within 14 d. Similarly targeting neural regeneration, Yang et al. [194] introduced an injectable silk fibroin/MXene conductive hydrogel functioning as a stem cell carrier for in vivo ES. This platform leveraged electrical cues to guide NSC fate, dramatically enhancing axonal extension while suppressing glial differentiation. In a TBI model, the hydrogel mitigated tissue loss, inhibited glial scar formation, and promoted in situ neuronal differentiation, thereby substantially accelerating motor function recovery.

**Fig. 19.**
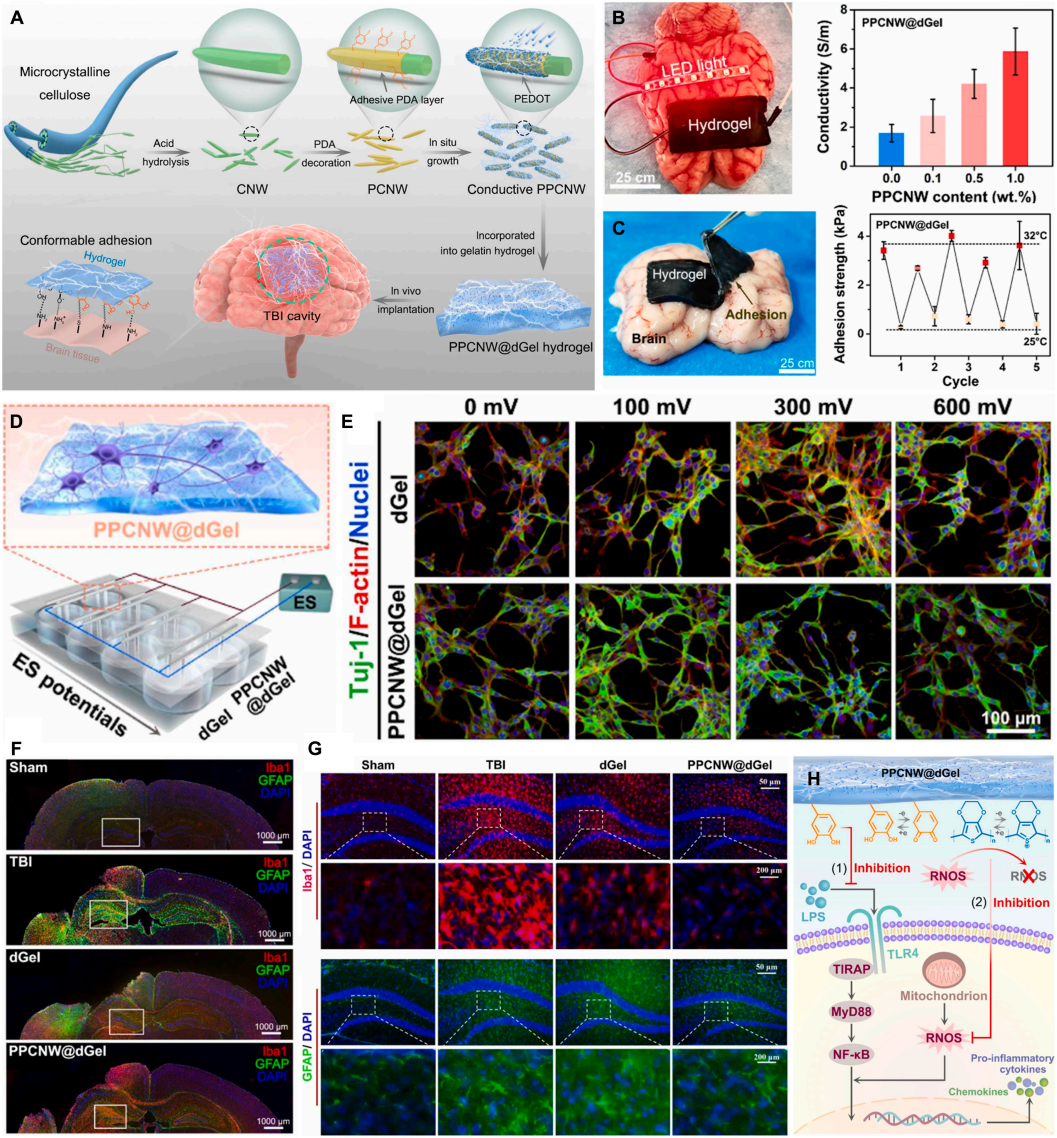
Adhesive-electrocoupling hydrogels for brain tissue repair. Reproduced with permission from [195]. Copyright 2023, Elsevier. (A) Schematic illustration of fabrication process of PPCNW@dGel adhesive-electrocoupling hydrogel. (B) The hydrogel connection to the circuit maintains the LED illumination and its conductivity. (C) Adhesion of the PPCNW@dGel adhesive-electrocoupling hydrogel to wet, fresh porcine brain tissue and the adhesive strength of the hydrogel when heated to 32 °C or cooled to 25 °C. (D) Schematic illustration of high-throughput ES of HT22 cells cultured on the PPCNW@dGel adhesive-electrocoupling hydrogel. (E) Immunofluorescence staining images of HT22 cells with Tuj-1 (green) and F-actin (red) on hydrogels under different ES conditions. (F) Representative fluorescence microscopy images of coronal brain sections after TBI. (G) Immunofluorescence images of Iba1 (red) and glial fibrillary acidic protein (GFAP) (green) cells in the dentate gyrus region of the hippocampus. (H) Schematic diagram of neuroprotective mechanism of PPCNW@dGel adhesive-electrocoupling hydrogel for TBI treatment via the TLR4–NF-κB signaling pathway.

To develop a nonpharmacological treatment strategy for TBI, Yan et al. [195] constructed a PPCNW@dGel adhesive-electrocoupling hydrogel (Fig. 19A), consisting of PEDOT-coated cellulose nanowhiskers (PPCNW) embedded in a dual-crosslinked gelatin (dGel) network. The incorporation of PPCNW enables the PPCNW@dGel hydrogel to conduct electrical signals and substantially enhances its overall conductivity (Fig. 19B). These hydrogels exhibit strong adhesion to brain tissue, preventing detachment. Moreover, their adhesion is temperature-responsive, activating only at physiological temperatures (Fig. 19C), which allows for convenient repositioning during surgery. The hydrogel’s softness and high adhesion at body temperature ensure intimate integration with surrounding brain tissue. When HT22 cells were seeded onto the PPCNW@dGel hydrogel and subjected to ES to simulate the brain’s endogenous electric field (Fig. 19D), the hydrogel demonstrated effective electrical coupling with differentiated neurons, promoting neuronal survival and neurite outgrowth (Fig. 19E). More importantly, in a TBI mouse model, PPCNW@dGel substantially alleviated neuroinflammation by inhibiting glial cell activation (Fig. 19F and G). The therapeutic mechanism is attributed to a dual-inhibition strategy. Catechol groups on the hydrogel surface interfere with lipopolysaccharide–Toll-like receptor 4 (TLR4) recognition, while the conductive components scavenge excess reactive nitrogen oxide species, collectively suppressing the downstream NF-κB inflammatory pathway (Fig. 19H). In summary, this adhesive-electrocoupling hydrogel presents a promising new paradigm for TBI treatment by integrating electro-coupling with immune modulation at the molecular level.

To address the long-term failure of traditional brain–computer interfaces caused by mechanical mismatch and foreign body responses, Wang et al. [103] developed an ultra-soft, adhesive-electrocoupling hydrogel. This hydrogel achieves mechanical compatibility with brain tissue through the introduction of dPEDOT NPs, which disrupt entanglements in the matrix polymer chains, allowing seamless integration with the brain’s complex surface geometry. Simultaneously, the catechol groups in dPEDOT NPs form an electron donor–acceptor system with PEDOT, in which PEDOT facilitates electron transfer to quinone groups, helping maintain sufficient catechol content. These abundant catechol groups suppress the immune response following implantation, effectively preventing fibrous capsule formation and neuroinflammation. As a result, this hydrogel enables long-term, stable EEG signal acquisition and communication while minimizing foreign body reactions. Table 1 lists the hydrogel materials, adhesion, conductivity, applications, and biological effects of adhesive-electrocoupling hydrogels used for tissue repair to make the information more intuitive.

**Table 1.** Adhesive-electrocoupling hydrogels for tissue repair and their characteristics

Hydrogel components	Adhesion	Conductivity	Applications	Biological effects	Ref.
GelMA, exosomes, PPy-TA	13.6 kPa for bone	~12 S/m	Bone regeneration	The “armor” protects exosomes from lysosomal degradation, thereby efficiently promoting osteoblast differentiation	[178]
Single-atom nanozyme, DA-HA, SO_3_^−^-HA	1.3 kPa for bone	/	Rheumatoid arthritis therapy	Eliminates joint inflammation and infiltration, improves joint lubrication in the long term, and repairs worn cartilage tissue	[180]
PGO, PHA, alginate, gelatin	Adhesion of BMSCs	1.6 S/cm	Periodontal bone regeneration in diabetes	It possesses immunomodulatory capabilities and ROS-scavenging functions, opens Ca^2+^ channels, and accelerates periodontal bone repair in the diabetic microenvironment	[105]
PEDOT-PSF, NaHS-encapsulated bovine serum albumin, alginate, gelatin	Adhesion of PDLSCs	13.12 S/m	Periodontal bone regeneration in diabetes	The continuous release of H2S recruits mesenchymal stem cells, delivers endogenous bioelectricity, promotes cell alignment, and increases Ca^2+^ influx	[182]
Nanoprobiotic, oxidized dextran, DHCA-grafted chitosan, gelatin	~28 kPa for bone	/	Diabetic periodontitis and its resulting cognitive decline	Restoring oral microbiome balance and regulating bone remodeling (inhibiting osteoclastization/activating osteogenics) alleviates anxiety-like cognitive impairment by disrupting the vicious cycle of the “oral–brain axis”	[185]
FGel-DA, SFMA, PDA-Fe-PEDOT	19 kPa for porcine skin	~50 S/m	Diabetic wound repair	Nanozymes possess peroxidase-like activity, exerting a sustained antioxidant effect under ES, protecting cells from oxidative stress	[188]
PDA-modified PEDOT, GelMA, DA, *Lycium barbarum* polysaccharide	Adhesion of RSC96	14 S/m	Diabetic wound healing and complication of depression	Conjugated electroacupuncture stimulation, through local “wound–brain” interaction, accelerates the repair of difficult-to-heal diabetic wounds and simultaneously alleviates depressive-like behaviors induced by diabetes	[12]
TA–Ag nanozyme, polyacrylic acid	26 kPa for porcine skin	21 S/m	Bioelectronics and skin wound repair	It can self-cure into an adhesive hydrogel with antibacterial properties, support the proliferation of C2C12, and promote skin wound healing	[106]
Conductive silk microfiber, silk fibroin	/	75 S/m	Bioelectronics and skin wound repair	Promoting wound healing in chronic diabetes by reducing inflammation and regulating the oxidative stress microenvironment.	[190]
PPEDOT NPs, GelMA, PGA	13 kPa for porcine hearts	~16 S/m	MI repair	It adheres firmly to dynamically moistened cardiac tissue, promoting the repair of MI areas by stimulating anti-inflammatory and calcium homeostasis pathways	[81]
PPCNW, gelatin	4 kPa for porcine skin	6 S/m	TBI repair	It can target neuroinflammation and neurological dysfunction, exerting anti-inflammatory effects and promoting neurogenesis	[195]
dPEDOT NPs, carrageenan, PDA, PAM	~9 kPa for porcine skin	42 S/m	Brain–machine interfaces	This ultrasoft hydrogel is highly biocompatible and features adhesive properties that ensure seamless integration with brain tissue. Furthermore, its inherent immunomodulatory capabilities render it exceptionally suitable for long-term implantable brain–machine interfaces	[103]

## Summary and Perspectives

This review has elucidated the pivotal role of polyphenol-based adhesive-electrocoupling hydrogels in bridging the biotic–abiotic interface. The unique architecture of these hydrogels leverages the catechol chemistry of polyphenols to achieve robust interfacial adhesion, which is the prerequisite for minimizing contact impedance and ensuring seamless tissue integration. Crucially, this intimate integration facilitates the effective coupling of the hydrogel with endogenous bio-electric fields. At the molecular level, this review highlighted the electron transfer-driven redox mechanism, where the reversible conversion between polyphenols (electron donors) and quinones (electron acceptors) orchestrates a dynamic charge transfer capability. This bio-inspired design not only creates a stable conductive pathway but also imparts the hydrogel with antioxidant and electroactive properties, establishing a sophisticated platform for tissue regeneration and bio-signal modulation.

While substantial progress has been made, the potential of adhesive-electrocoupling hydrogels extends far beyond current paradigms. Future research should focus on 3 strategic directions to maximize their impact on biomedicine. First, generalizing the electron transfer-driven adhesive-electrocoupling paradigm to broader material systems remains a critical future direction. While polyphenol-based hydrogels have demonstrated the efficacy of the phenol–quinone redox cycle in achieving simultaneous tissue adhesion and electrocoupling, this mechanism should be viewed as a generalizable blueprint rather than an isolated case. Second, future developments should transition from passive sensing to active modulation by incorporating exogenous electric fields. Currently, most applications focus on coupling with endogenous bioelectricity. A critical frontier lies in amplifying biological recovery by integrating these adhesive hydrogels with exogenous electric field. Due to their superior interfacial integration, these hydrogels can respond efficiently to external ES. Furthermore, a critical limitation arises in senescent tissues, where endogenous bioelectric fields are often substantially attenuated or obstructed due to physiological barriers and cellular quiescence [2,196]. Therefore, a pivotal frontier lies in utilizing these adhesive-electrocoupling hydrogels to deliver exogenous ES to reactivate dormant repair pathways. Finally, future strategies should explore “closed-loop” systems where the hydrogel not only senses bio-signals via the polyphenol-mediated interface but also delivers programmed ES to guide cell migration, synchronize tissue contraction, or accelerate wound healing. Enhancing the bio-utilization of electricity requires optimizing the hydrogel’s impedance matching with biological tissues to prevent signal attenuation, thereby transforming the material from a passive scaffold into an active electro-therapeutic device.

Although adhesive-electrocoupling hydrogels exhibit promising therapeutic potential, many current formulations rely on nonbiodegradable metal-based conductive nanomaterials, carbon nanomaterials, and conductive polymers. The long-term retention of these materials within the physiological environment can precipitate adverse reactions. These complications include the leakage of toxic metal ions, the accumulation of nonclearable degradation products in metabolic organs, and the induction of chronic inflammatory responses or severe fibrotic capsule formation. Consequently, future research should prioritize the development of fully biodegradable or renally clearable conductive components. In addition, establishing standardized, comprehensive in vivo assessment protocols to rigorously evaluate the chronic toxicity, immune responses, and metabolic pathways of these electroactive materials is crucial for their safe clinical translation. Furthermore, the integration of cutting-edge technologies, particularly artificial intelligence (AI), represents a major frontier in the development of adhesive-electrocoupling hydrogels [197]. Traditional hydrogel formulation optimization processes, which rely on trial and error, inherently suffer from protracted development cycles and inefficiency. In contrast, performance screening driven by AI can accurately predict the optimal ratios of polyphenols, conductive fillers, and polymer backbones, facilitating the customized control of their mechanical, adhesive, and electrical properties. The application of AI to the rational and precise design of these electroactive biomaterials is expected to substantially accelerate their translation from laboratory prototypes to personalized clinical therapies.

Despite the promising therapeutic efficacy observed in preclinical models, translating polyphenol-based adhesive-electrocoupling hydrogels into clinical practice necessitates addressing 2 critical hurdles. First, the rigorous evaluation of long-term stability and biocompatibility remains the primary prerequisite for clinical entry. Current research predominantly relies on in vitro assays and short-term animal models, which fail to fully recapitulate the complex, dynamic, and chronic physiological environments of humans. A transition from small-animal efficacy studies to long-term evaluations in large-animal models is essential to bridge the gap between laboratory prototypes and viable clinical products. Second, addressing patient heterogeneity is imperative for realizing precision medicine. Most existing adhesive-electrocoupling hydrogels adopt a noncustomized design strategy, overlooking the substantial physiological variations arising from different ages and disease severities. For instance, the electrical properties and healing capacities of aged or severely fibrotic tissues differ drastically from those of young, healthy tissues. This variability suggests that a standardized hydrogel formulation may not yield consistent therapeutic outcomes across diverse patient populations. Consequently, developing adaptive or personalized hydrogel systems that can be tailored to the specific microenvironmental requirements of different patient stratifications represents a formidable yet necessary challenge for future research.

Beyond localized tissue reconstruction, the therapeutic paradigm of adhesive-electrocoupling hydrogels is expanding to encompass complex inter-organ interactions. Peripheral tissue injuries frequently exert systemic effects on the central nervous system, increasing susceptibility to anxiety, depression, and cognitive impairment [198]. Consequently, a pivotal frontier for future research lies in developing hydrogels that not only facilitate in situ tissue repair but also mitigate these secondary central nervous system complications, effectively bridging the gap between local healing and central nervous system well-being. In summary, adhesive-electrocoupling hydrogels exhibit immense potential within the realm of tissue repair therapy. Addressing current challenges and further elucidating the underlying repair mechanisms are expected to accelerate the clinical translation and widespread application of these hydrogels.
